# Physical Mechanisms of an Unconventional Green Fluorescent Protein Indicator for Chloride

**DOI:** 10.1021/acs.jpcb.5c08244

**Published:** 2026-03-05

**Authors:** Mfon V. Sunday, Ke Ji, Derik A. Adams, Weicheng Peng, Sheel C. Dodani, Alice R. Walker

**Affiliations:** † Department of Chemistry, 302573 Wayne State University, Detroit, Michigan 48202, United States; ‡ Department of Chemistry and Biochemistry, 12335 The University of Texas at Dallas, Richardson, Texas 75080, United States; § Department of Biological Sciences, The University of Texas at Dallas, Richardson, Texas 75080, United States

## Abstract

Fluorescent proteins bearing an intrinsic tripeptide chromophore exhibit diverse, tunable photophysical features that are exceptional for biosensing applications. However, atomic-level details of these sensing mechanisms are obscured experimentally, particularly as protein motions, including the chromophore, cannot be divined from the structure alone. Molecular dynamics (MD) simulations can bridge this gap, providing a landscape of global and local protein motions with resolution to key amino acids connected to function and potential for engineering. In this study, we uncover that the green fluorescent protein from the jellyfish *Clytia gregaria* (cgreGFP) is sensitive to anions, including chloride, bromide, iodide, and nitrate, with a combination of theoretical and experimental investigations. Constant pH molecular dynamics (CpHMD) simulations reveal a coordinated entry of all four anions into an unconventional binding cavity near the chromophore. Photophysical measurements of the wild-type protein confirm this behavior and demonstrate that anion binding tunes the chromophore equilibrium, resulting in a turn-off fluorescence response at acidic pH with the affinity trend iodide > nitrate > bromide > chloride. Finally, targeted mutagenesis of the anion entry pathway emphasizes the guiding force of theory to understand cgreGFP-like indicators and beyond.

## Graphical abstract

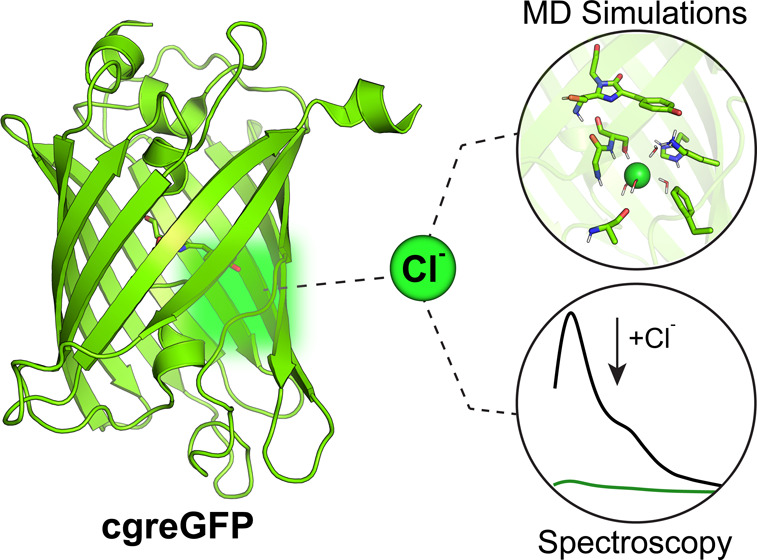

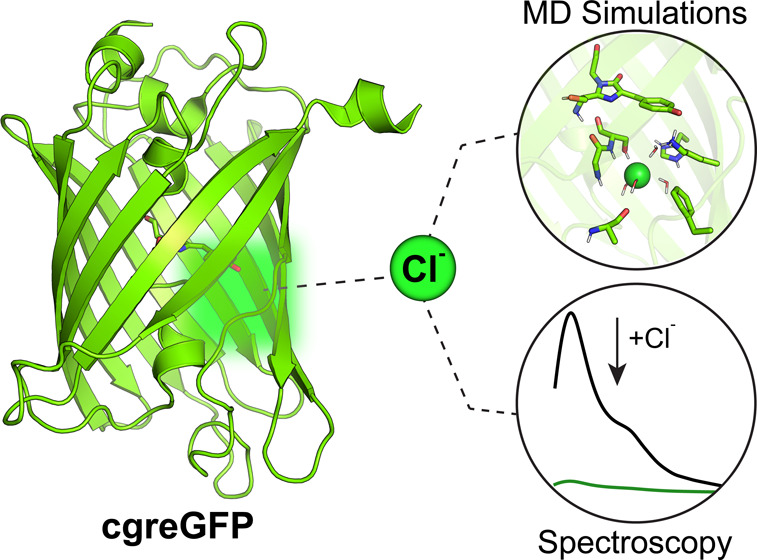

## Introduction

Since the discovery of the green fluorescent protein from the jellyfish *Aequorea victoria* (avGFP) more than 50 years ago, new naturally occurring fluorescent proteins have continued to be unearthed from diverse ecological niches.^1–3^ While the overall β-barrel architecture is conserved around the tripeptide chromophore, sequence level differences give rise to unique photophysical properties.^4^ As a result, fluorescent proteins have become not only the subject of intense biochemical investigations but also the focus of engineering efforts to unlock biotechnological potential, particularly for biosensing.^5–7^ To accelerate this path forward and deepen mechanistic insights, theoretical investigations of fluorescent protein motion have an important role to play, yet they remain underutilized.^8–12^ This gap becomes apparent when considering the protein sequence alongside its environment (e.g., hydration, molecular oxygen, and analytes for biosensing applications). The interconnectedness of these two facets shapes global and local protein motions, including those at the chromophore level, ultimately manifesting in photophysical behavior and, thus, sophisticated sensing mechanisms. In this pursuit, we have a growing collaborative program that partners theory and experiment to unravel the molecular drivers of anion sensing in fluorescent proteins for ultimate applications in cellular imaging.^13–15^

The earliest anion-sensitive avGFP variants were reported more than 25 years ago.^16–18^ One of the most widely characterized and utilized engineered variants is YFP-H148Q (Figure 1A).^19,20^ Halides and oxyanions can enter the β-barrel through a solvent channel between the β7 and β10 strands and bind in a cavity in the proximity of the chromophore.^13,20,21^ The solvent channel is formed by distortions in the hydrogen bonding network around the chromophore along the β7 strand at the gate post (145/148) and β-bulge (146/147) segments.^20^ The histidine to glutamine mutation at position 148 likely tunes the solvent channel opening, enhancing anion access and binding affinity to the cavity.^20^ The coordinating amino acids Q69, R96, Q183, and Y203 line the cavity, where the latter residue forms a critical π-π stacking interaction with the chromophore, which is further networked to a proton relay with water, S205, and E222.^20^ Anion binding to avYFP-H148Q and related variants shifts the chromophore protonation state from the fluorescent phenolate to the nonfluorescent phenol, translating into a turn-off fluorescence response (Figure 1B).^20^ Indeed, protein engineering has been used to explore and expand the scope of avYFP-H148Q with adaptations of the parent avGFP scaffold to create E^2^GFP-based biosensors with a distinct static fluorescence quenching mechanism.^22–33^ More recently, we and others have looked to unearth GFP-like scaffolds from existing sequence space and unlock their anion sensing potential revealing unprecedented features and mechanisms as compared to YFP-H148Q. Included are phiYFP, mNeonGreen, mBeRFP, GFPxm163, SulfOFF, ChlorON-1/2/3, Thyone, and ChlorOFF.^13–15,34–40^

**Figure 1:**
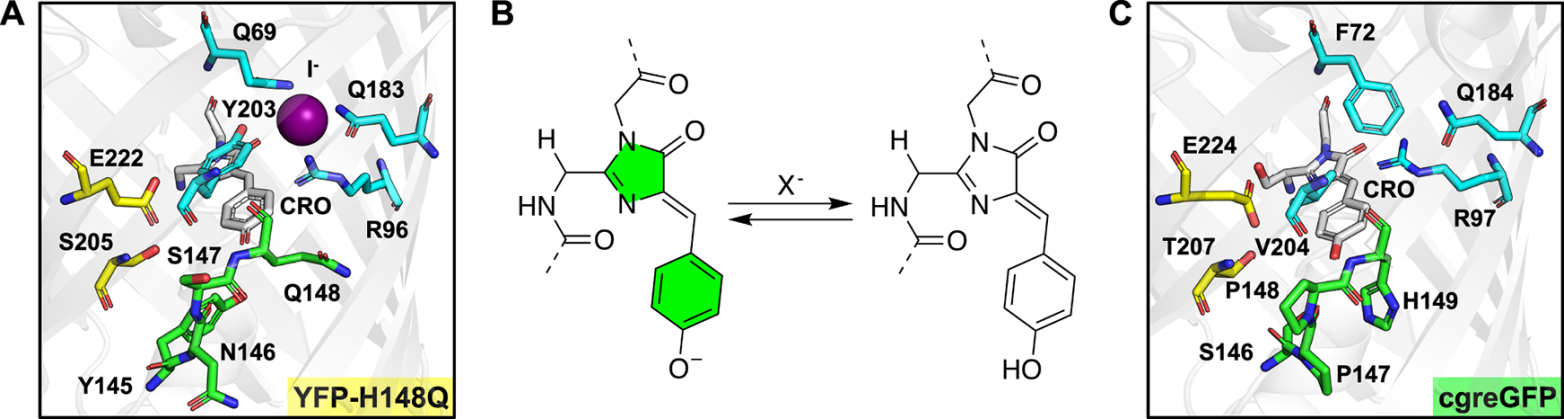
(A) X-ray crystal structure of the anion-sensitive GFP derivative YFP-H148Q (PDB ID: 1F09). The residues in the anion binding pocket (cyan), gate post and β-bulge region (green), and in proximity to the chromophore (yellow) are shown. (B) Anion-dependent tuning of the chromophore equilibrium and fluorescence state where X^–^ can be chloride, bromide, iodide, or nitrate. (C) X-ray crystal structure of the bioinformatically identified cgreGFP (PDB ID: 2HPW). The chromophore and residues homologous to those in panel (A) are shown. Abbreviation: CRO, chromophore.

Across all of these systems, static structural insights combined with photophysical characterization have shaped our understanding of anion sensing-mechanisms and guided engineering strategies. This has enabled the optimization of key functional properties, including anion specificity, spectral tuning, response type (i.e., turn-on versus turn-off), and pH sensitivity. While tunable, the relationships among sequence, structure, and function are often obscure. However, incorporating molecular dynamics (MD) simulations and simulating physical features connecting the chromophore’s behavior to the protein scaffold adds a dynamic dimension, revealing molecular level details that are often inaccessible through experimental techniques alone.^8–12,41^ This enables researchers to predict or rationalize experimental outcomes, challenging prior assumptions and generating new insights to inform biosensor design. Specifically for our anion biosensors (i.e., SulfOFF, ChlorONs), these approaches have revealed ion association with the protein surface, entry pathways, and stabilization in the final binding cavity, alongside conformational motions of residues and the chromophore in response to anion recognition.^13–15^ Building on the success of our integrated process, we envisioned that MD simulations could be leveraged and combined with bioinformatic mining prior to experimental validation to rapidly identify anion-sensitive fluorescent proteins. As a proof-of-concept, we showcase this possibility by uncovering the anion sensing potential of the green fluorescent protein from the jellyfish *Clytia gregaria* (cgreGFP).

## Methods

### Bioinformatics to Identify cgreGFP

A sequence alignment of 806 members from the green fluorescent protein family (PF01353) was generated and downloaded from the protein family (Pfam) database in February 2019. Using Biopython, 71 members were identified with at least two residues matching the binding pocket of YFP-H148Q (PDB ID: 1F09).^20^ From this, members with different residues at the Q69 and Y203 positions were further reviewed. Of these, proteins with hydrophobic residues, namely, F69 and V203, in the putative anion binding pocket were identified. The GFP from *Clytia gregaria* (cgreGFP, UniProt ID: D7PM05) was selected for further testing because it was previously structurally characterized (PDB ID: 2HPW).^42,43^

### Molecular Dynamics

The initial structure of the cgreGFP protein was obtained from the Protein Data Bank (PDB ID: 2HPW).^43^ We then used the MolProbity web server to add hydrogen atoms, optimizing hydrogen bonding networks.^44^ To prepare for constant pH molecular dynamics (CpHMD) as implemented in Amber20,^45^ we manually changed all ASP, GLU, and HIS residues to AS4, GL4, and HIP to enable their sampling as titratable residues during the simulation.^46–48^ Custom parameters for nitrate and the embedded chromophore (SYG) were generated using the Autoparams web server.^49^ The system was solvated with the TIP3P water model, leaving a pad of a minimum of 12 Å of water between the protein and the box edge.^50^ The protein was parametrized with the ff14SB Amber force field, and the inputs were generated with the tleap module of AmberTools22.^51^ All simulation input files and force field parameters, as well as key outputs, are deposited in the Zenodo repository (DOI: 10.5281/zenodo.15023056). The initial protonation states of the titratable residues were set randomly, and each system was run in triplicate for 400 ns, with the exception of our mutated system, as detailed further below.

We ran several types of systems in order to probe the cgreGFP’s potential ion response and mechanism. First, to find the anion binding pathway for each ion type (chloride, bromide, iodide, nitrate), we set up free diffusion of the anions around the apo cgreGFP protein similarly to previous work.^13^ We determined the number of sodium ions and the corresponding anions of interest needed for a neutral system with an ionic strength of 300 mM.^52^ For example, for nitrate, we randomly added 67 nitrate ions and 75 sodium ions around apo cgreGFP. Each free diffusion system was set to run at pH 5 and 7, though we only observed ion binding for trajectories run at a pH of 5.

Simulations using GPU-accelerated CpHMD incorporated smooth particle mesh Ewald for nonbonded forces.^48,53^ Each system was minimized and heated to 300 K, followed by structural and density equilibration in the *NPT* ensemble for 4 ns. For the production simulations, the pH and salt concentration were set to 5/7 and 0.3 M, respectively, while the protonation state was configured to change every 100 steps. The simulation employed periodic boundary conditions, with the nonbonded interactions cutoff distance set to 10 Å. Using a 2 fs time step, each trajectory was run for 500 ns. The temperature was controlled through Langevin dynamics with a collision frequency set to 5.0 ps^–1^, and covalent hydrogen bonds were constrained using the SHAKE algorithm.^54,55^

After determining the binding pathway and pocket from the free diffusion simulations, we ran additional systems set at a pH of 5 to test various details of cgreGFP–anion complex stability. Iodide was the only anion that did not proceed to the final binding pocket observed for all of the other anions. We therefore ran an additional system for each anion, where it was manually placed in the observed binding pocket at the beginning of the simulation to determine that each anion was stable. Additionally, we simulated the cgreGFP–chloride system with chloride placed in the proposed binding pocket from the original bioinformatics. We also ran an additional free diffusion trajectory for chloride with the dimer system and compared it to that of the monomer. In this case, the results did not differ between the monomer and the dimer systems. Finally, we performed *in silico* mutagenesis to suggest a mutant for experimental validation, where the bound chloride structure was mutated in PyMOL 3.0.^56^ All of these simulations used the same simulation parameters described above except for the mutagenesis simulations, which were truncated at 20 ns due to the speed with which the anion left the binding pocket.

The cpptraj module from Amber22 was employed to analyze the simulation trajectories, including the root-mean-square deviation (RMSD), root-mean-square fluctuation (RMSF), hydrogen bond occupancies, radial distribution functions around relevant anions, and solvent-accessible surface area (SASA). All molecular visualizations, structural depictions, and trajectory analyses were created using VMD 1.9.4 and PyMOL 3.0.^56,57^

### General Experimental Information

All reagents and supplies used in this study were acquired from Sigma-Aldrich, Thermo Fisher Scientific, Thomas Scientific, VWR, USA Scientific, or Research Products International, except where noted.

### Plasmid Design and Cloning

The cgreGFP gene was synthesized and codon-optimized for expression in *Escherichia coli* (GenScript). The gene was cloned in a pET-28a­(+)-TEV vector between the NdeI and *Bam*HI restriction sites resulting in an in-frame N-terminal polyhistidine tag and a C-terminal stop codon. The cgreGFP-H149A mutant was generated as follows using commercially available kits and procedures. The mutation was introduced into the cgreGFP plasmid by using site-directed mutagenesis primers with the Phusion Master Mix (New England Biolabs). The resulting PCR product was treated with Dpn1 (New England Biolabs), followed by separation with agarose gel electrophoresis and extraction (Zymo Research). Approximately 100 ng of DNA was mixed with the Gibson Assembly Master Mix (New England Biolabs) and isolated using a DNA Clean and Concentrator kit (Zymo Research).

### Protein Expression and Purification

*E. cloni* EXPRESS BL21 (DE3) electrocompetent cells (Lucigen) were transformed with the cgreGFP-pET-28a­(+)-TEV plasmid, and the cgreGFP protein was expressed and purified as previously described with the following two modifications.^13^ The protein expression was carried out at 22 °C, and the purified protein was dialyzed with 20 mM MOPS buffer at pH 7.0 with 50 mM NaCl using a Slide-A-Lyzer Dialysis Cassette according to manufacturer’s instructions. This procedure was carried out for two different colonies.

For cgreGFP-H149A, *E. cloni* 10G Elite Electrocompetent Cells (Lucigen) were transformed with the DNA isolated with the DNA Clean and Concentrator kit described above. The plasmid DNA was isolated using QIAprep Spin Miniprep Kit according to the manufacturer’s instructions and prepared for Sanger sequencing (Eurofins) to identify a positive clone. *E. cloni* EXPRESS BL21 (DE3) electrocompetent cells were transformed with the sequence verified cgreGFP-H149A-pET-28a­(+)-TEV plasmid, and the cgreGFP-H149A protein was expressed as described above for the wild-type protein.

The purity and concentration of each protein batch were determined using our previously reported methods.^13^ The extinction coefficient for the full-length protein including the polyhistidine tag was determined to be 37,360 M^–1^ cm^–1^ using the ProtParam program in ExPASy.^58^ Following this, the protein samples were aliquoted and frozen at −20 °C until further use.

### General Spectroscopy

For all spectroscopic characterizations, except for the extinction coefficient and quantum yield described below, the following settings were used on a plate reader (Spark, Tecan). Measurements were carried out at room temperature (24–26 °C) in a 96-well UV-Star microtiter plate (Greiner Bio-One). For each protein batch, three technical replicates were carried out for all measurements. Absorbance spectra were collected from 350 to 550 nm (2 nm step size, 3.5 nm bandwidth). The following settings were used for cgreGFP. For the excitation provided at 394 nm (5 nm bandwidth), the emission was collected from 430 to 650 nm (5 nm step size, 5 nm bandwidth, 30 flashes, 80 gain). For the excitation provided at 470 nm (5 nm bandwidth), the emission was collected from 490 to 650 nm (5 nm step size, 5 nm bandwidth, 30 flashes, 80 gain). The following settings were used for cgreGFP-H149A. For the excitation provided at 384 nm (5 nm bandwidth), the emission was collected from 430 to 650 nm (5 nm step size, 5 nm bandwidth, 30 flashes, 80 gain). For the excitation provided at 460 nm (5 nm bandwidth), the emission was collected from 480 to 650 nm (5 nm step size, 5 nm bandwidth, 30 flashes, 80 gain).

### Chromophore p*K*_a_ Determination

Frozen aliquots of each protein batch were thawed and diluted 200-fold to a final protein concentration of ∼3 μM for cgreGFP and ∼2 μM for cgreGFP-H149A in 20 mM citric acid/sodium citrate buffer from pH 3.5 to 5.5, 20 mM MES buffer from pH 6 to 6.5, 20 mM sodium phosphate buffer from pH 6.5 to 8.0, containing 0 or 100 mM NaCl. Three 200 μL of each diluted sample for each protein batch were transferred to a microtiter plate. We note that after dilution, all samples retained ∼0.25 mM NaCl.

For each well, the averaged absorbance intensity at 470 nm for cgreGFP or 486 nm for cgreGFP-H149A was plotted versus the pH; the averaged emission intensity at 495 nm for cgreGFP or 500 nm for cgreGFP-H149A was normalized and plotted versus the pH. The normalized emission intensity could not be fitted to the Henderson–Hasselbalch equation to determine the p*K*_a_. As such, the p*K*_a_ was estimated based on the pH at which 50% of fluorescent signal was retained. The p*K*_a_ values are reported as the average from both protein batches.

### Anion Titrations

Frozen aliquots of each purified protein were thawed and diluted 200-fold to a final protein concentration of ∼3 μM for cgreGFP in 20 mM citric acid/sodium citrate buffer at pH 5 containing varying concentrations of sodium chloride, bromide, iodide, nitrate, and gluconate. Three 200 μL of each sample were transferred to a microtiter plate (Greiner Bio-One). We note that after dilution all samples retained ∼0.25 mM NaCl. The apparent dissociation constant (*K*_d_) using the following equation as previously described.^13^

$$F_{\mathrm{obs}}=\left(\frac{{\left[X^{-}\right]}^{*}\left(F_{\max}-F_{\min}\right)}{\left(K_{d}+\left[X^{-}\right]\right)}+F_{\min}\right)$$

where *F*_obs_ is the average fluorescence intensity at 500 nm for each concentration tested and *F*_min_ and *F*_max_ are the average fluorescence intensities at 500 nm (λ_ex_ = 470 nm) in the presence of 0 and 100 mM anion. For each anion, the *K*_d_ values from both protein batches are reported as the average with the propagated standard deviation.

For the anion selectivity bar graphs, frozen aliquots of each purified protein were thawed and diluted 200-fold to a final protein concentration of ∼3 μM for cgreGFP or ∼2 μM for cgreGFP-H149A in 20 mM citric acid/sodium citrate buffer at pH 5 containing 0, 10, or 100 mM sodium chloride, bromide, iodide, nitrate, or gluconate. Three 200 μL aliquots of each sample were transferred to a microtiter plate. We note that after dilution all samples retained ∼0.25 mM NaCl. The fold-change (*F*_*f*_/*F*_*i*_) was calculated where *F*_*i*_ is the emission intensity at 500 nm in the absence of any anion, and *F*_*f*_ is the emission intensity at 500 nm in the presence of 10 or 100 mM anion. The fold-changes from both protein batches are reported as the average with the propagated standard deviation.

### Protein Extinction Coefficients and Quantum Yield Determination

The extinction coefficients (*ε*) of each protein batch were determined using a previously described procedure with the following modifications.^13^ Frozen aliquots of cgreGFP were thawed in a fridge and diluted 200-fold to a final protein concentration of ∼3 μM in 20 mM citric acid/sodium citrate buffer at pH 5 containing 0 or 100 mM sodium chloride. Three 200 μL aliquots of each sample were transferred a microtiter plate. We note that after dilution all samples retained ∼0.25 mM NaCl. The absorbance intensities at 280, 394, and 485 nm were used to determine the extinction coefficients at 394 and 485 nm.^58^ The extinction coefficients from both protein batches are reported as the average with the propagated standard deviation.

The quantum yield (Φ) of each protein batch was determined using a previously described procedure with the following modifications.^13^ The protein samples were prepared following the same procedure in extinction coefficient experiments above. The absorbance spectra were collected from 250 to 650 nm (5 nm step size, 3.5 nm bandwidth). Excitation was provided at 470 nm (5 nm bandwidth) and 450 nm (5 nm bandwidth), and the emission was collected from 490 to 650 nm and from 470 to 650 nm, respectively (2 nm step size, 5 nm bandwidth, 30 flashes, 80 gain). The ratio of the integrated areas from 490 to 650 nm for λ_ex_ = 470 nm and from 470 to 650 nm for λ_ex_ = 450 nm was used to estimate the integrated area from 470 to 490 nm for the emission spectra of λ_ex_ = 470 nm. Linear plots were generated by plotting the integrated area that was estimated from 470 to 650 nm for λ_ex_ = 470 nm versus the absorbance intensity at 470 nm and further processed to calculate the quantum yield versus fluorescein, which was cross-referenced to Coumarin-153.^59,60^ The quantum yields from both protein batches are reported as the average with the propagated standard deviation.

## Results and Discussion

### Bioinformatics

To identify cgreGFP, we applied a bioinformatic mining approach, drawing focus to the anion coordination sphere of YFP-H148Q (Figure 1A). From the 806 members in the GFP protein family, 71 members were aligned and filtered out, bearing at least two of the four binding cavity residues (Table S1). Conventionally, the anion binding cavity lies above the chromophore close to the phenolate moiety (Figure 1A), though the composition can vary.^36^ While a high degree of conservation was apparent at R88 and Q183, clear diversity emerged at Q69 and Y203 (Table S1). For fluorescent proteins in general, the arginine at position 88 is functionally essential for chromophore maturation and stabilization.^61^ Similarly, the glutamine at position 183 is in close proximity to chromophore, with no to little reported tolerance for substitution.^61,62^ We note in anion-sensitive GFPs, Q183A can dramatically enhance anion sensitivity at the cost of overall brightness.^24^ Looking to position 69, the distribution of possible amino acids ranged from glutamine to leucine, lysine, methionine, phenylalanine, serine, and tyrosine. Of these, only glutamine, lysine and methionine, in addition to threonine and histidine, are known to influence anion coordination.^24,27,40^ Aside from tyrosine at position 203, asparagine, cysteine, phenylalanine, leucine, serine, threonine, and valine were identified. Indeed, Y203 is linked to anion sensing potential with rarer substitutions of arginine, isoleucine, and leucine.^20,34,36,40^

With the R88 and Q183 amino acids fixed, we next considered the polarity of the amino acids at positions 69 and 203 in the context of each other. One combination of nonpolar amino acids stood out—namely, F69 and V203. At first, F69 was not intuitive, but the potential for anion−π or anion–C–H, along with water-mediated, interactions could not be ruled out.^63–65^ We rationalized that this in combination with a noncoordinating V203 would force R88 and Q183 to compensate for coordination, resulting in an anion-sensitive fluorescent protein. Such a scenario seemed plausible when looking at the X-ray crystal structure of one of the proteins cgreGFP (UniProt ID: D7PM05), which revealed two key features (Figure 1C).^43^ One, the proximity of the putative binding cavity affects the tyrosine-centered chromophore generated from the cyclization of serine, tyrosine, and glycine. Two, the presence of an ionizable histidine residue at position 149 on the β7 strand at the critical gate post position—the equivalent of Q148 in YFP-H148Q (Figure 1A). In the context of the field, the naturally occurring cgreGFP has been isolated as a recombinant protein for spectroscopic characterization, engineered, and the subject of MD simulations, but it has yet to be evaluated as an anion-sensitive fluorescent protein.^42,43,66,67^

### Molecular Dynamics: Free Diffusion and Binding Pathways of Anions

To test this possibility, we first performed explicit solvent constant pH molecular dynamics (CpHMD) simulations of cgreGFP and explored its interactions with anions at pH 5 and 7 (see Methods).

CpHMD accounts for pH-dependent protein motions and provides insights into relative anion binding affinities at atomic resolution.^47,48^ Anion binding was observed at pH 5, but not pH 7, which is consistent with other fluorescent proteins such as mNeonGreen.^36^ While anion binding is needed for photophysical responsiveness, it does not necessarily follow that the fluorescence of the chromophore will be affected to generate an optical change. For example, StayGold, a photostable GFP that was crystallized with a bound chloride, is not sensitive to anions.^68^ We therefore looked to additional factors beyond binding that could affect the photophysics of the chromophore. These included structural changes, such as twisting of the chromophore, rearrangements in binding pocket residues, shifts in residue protonation states, and altered solvation within the β-barrel.

The protein was simulated in a dynamic environment, allowing the bulk solvent and anions to freely access the β-barrel. We specifically selected anions known to interact with fluorescent proteins, including chloride, bromide, iodide, and nitrate.^39,69^ During our simulations, all four anions were associated primarily with H145 on strand β7, as well as ​Y144 on strand β8, both of which can face the bulk solvent or the interior of the β-barrel depending on the protein conformation (Figure 2). After surface association, we captured the free diffusion of chloride, bromide, and nitrate, but not iodide, into the β-barrel through a common pathway, albeit at different time scales. An overview of this common pathway, illustrated by chloride as an example, is shown in Figure 2. The anions entered the transient opening between residues F208 and S146 on β-barrel strands 7 and 8 caused by the rotation of Y144, which led to a positively charged tunnel within the β-barrel, ending at our final observed binding cavity (Figure 3, Supplemental Movie 1). While anions can also associate with other positively charged regions of the protein surface, such as R181, they do not enter the β-barrel from other surface positions (Figure 2B, Figure 3).

**Figure 2:**
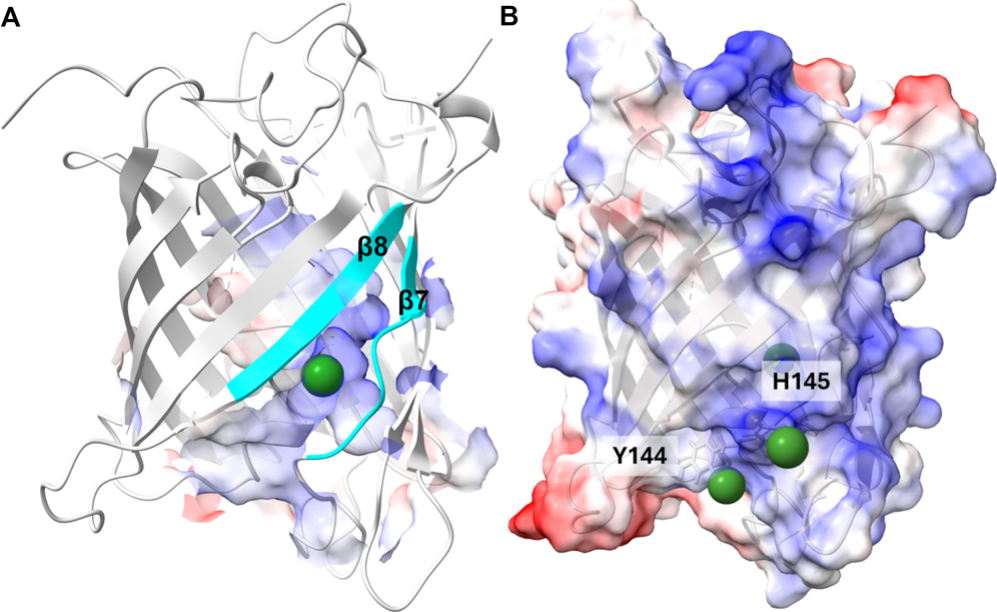
(A) The overall protein structure (gray) is shown with the inner surface of the protein colored by positive (blue) and negative (red) charge. The opening β7 and β8 strands are labeled and highlighted in cyan with a representative chloride ion (green sphere). (B) The outer surface of the protein structure is colored by charge with associated residues Y144 and H145 labeled.

**Figure 3:**
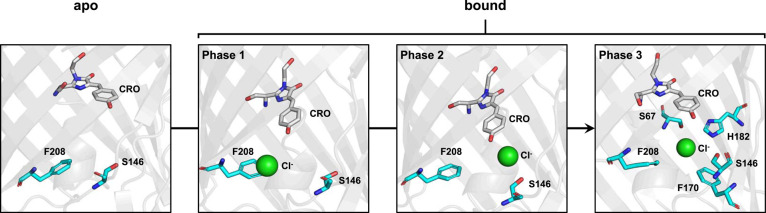
Representative snapshots from the simulated anion entry pathway for chloride in cgreGFP. The overall protein structure is shown with the chromophore (gray) and key residues (cyan) within 4 Å of the anion. Abbreviation: CRO, chromophore.

Chloride, bromide, and nitrate consistently interacted, in different combinations, with a common set of residues (≤4 Å) that we defined as the anion binding cavity within the β-barrel: L66, Y64, S67, R97, F101, K168, and H182 (Figure 4). This result was unexpected based on our prior prediction from YFP-H148Q in Table S1 as the position of this new, unconventional binding cavity was shifted ∼8–9 Å beneath the chromophore. Iodide, in contrast, displayed distinct behavior. It remained stable when it was manually placed in the same binding cavity over the course of the 500 ns simulation. However, in one free diffusion replicate, iodide followed an alternative entry pathway near residues S146 and K168 and did not fully occupy the anion binding cavity (Figure S1). Despite differences among the anions, the chromophore consistently shifted in position but retained its planarity and overall motion upon anion binding (Figure S2), suggesting two key features of the sensing mechanism. First, this observation indicated that nonradiative decay via isomerization is unlikely to differ sufficiently before and after anion binding to the point of generating a turn-off response through isomerization alone, though we cannot completely preclude this possibility.^10,70^ Second, in our previous work, we found that changes in the phenolate ring position can be associated with a turn-off fluorescence response, wherein a proton relay network leads to chromophore protonation and conversion to the nonfluorescent phenol form.^13^

**Figure 4:**
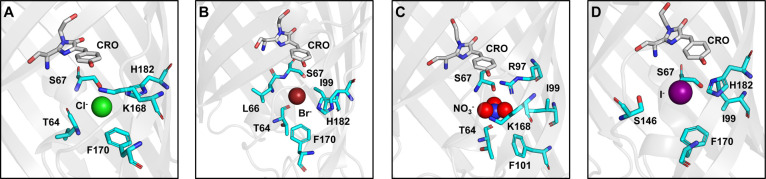
Representative snapshots from the simulated anion binding cavities for (A) chloride, (B) bromide, (C) nitrate, and (D) iodide. The overall protein structure is shown with the chromophore (gray) and binding pocket residues (cyan) within 4 Å of the anion. Abbreviations: CRO, chromophore.

### Molecular Dynamics: Equilibration and Protein Stability Analysis

To evaluate the structural stability of the protein in the presence of the anions, we calculated the root-mean-square deviation (RMSD) of the heavy protein backbone atoms for each trajectory frame relative to the initial conformation over a 500 ns period from the free diffusion simulations (Figure S3). Protein conformational responses were observed for all four anions upon binding. During the first 50 ns, all systems had increasing RMSD values of up to 3 Å. This is in line with an initial equilibration phase as a system adapts to the simulation environment. The chloride, bromide, and nitrate-bound systems stabilized within an RMSD range of 3.0–4.0 Å compared to the initial frame, consistent with a moderate level protein fluctuation in a stable simulation environment. Upon closer inspection, the RMSD of the chloride-bound system was higher (∼3.5 Å) than those of the bromide- and nitrate-bound systems (∼3.0 Å). This suggested that the protein was more conformationally restrained in the presence of bromide and nitrate.

However, the iodide-bound system stabilized at a higher RMSD than chloride (∼4.0 Å), indicative of increased conformational flexibility, potentially arising from weaker electrostatic interactions or distinct hydration dynamics of each anion.^69^ Overall, the RMSD differences underscore the anion-specific effects on the protein structure, reflecting the differences in the binding modes and strengths. Nonetheless, the RMSDs indicated that the protein remained structurally stable throughout the MD simulations.

### Molecular Dynamics: Binding Cavity

Next, we examined the composition and spatial arrangement of the seven binding cavity residues to determine the importance of specific residues within the β-barrel. To do so, we analyzed all anion-protein contacts at ≤4 Å. The positively charged residues R97 and H182, and at least three polar residues, albeit varied in identity and combination, were consistently present in each coordination sphere, further stabilizing the anion-protein complex (Figure 4). Each anion interacted with slightly different residues in the binding cavity, correlating with their relative sizes. The ionic radii can be ranked from smallest to largest as follows: nitrate ≈ chloride < bromide < iodide.^69^ This trend was clearly exemplified by the two extremes: nitrate interacted more closely with R97 and K168 in the binding cavity (Figure 4C), while iodide remained closer to S146 on the opposite side (Figure 4D). Nitrate adopted different orientations, given its polyatomic nature, and formed favorable hydrogen bonding interactions, particularly with R97 and K168, driving the displacement of water molecules (Figure S4). On the other hand, iodide remained close to the entry pathway described above, making contacts with Y166 and H182 (Figure S1).

### Molecular Dynamics: Solvation Effects

Finally, to complete the *in silico* picture, we probed the role that the solvent could play in anion recognition by cgreGFP. To do so, we first calculated the radial distribution function (RDF) of water molecules surrounding each anion as it approached the binding cavity (Figure 5). Overall, the first solvation shell peaks clustered within a narrow range from ∼3.5–3.8 Å, but the magnitude and distribution of the probability differed among the anions, reflecting distinct hydration profiles. Chloride (ionic radius: 1.81 Å) and bromide (ionic radius: 1.96 Å) exhibited the highest peak intensities indicating a higher degree of hydration, with bromide’s first solvation shell RDF peak shifted by ∼0.1 Å due to its larger ionic radius (Figure 5).^69^ In fact, trajectory analysis further confirmed the presence of at least four water molecules in the first solvation shell coordinating with both anions at any given time (Figure 6). In contrast, nitrate (ionic radius: 2.64 Å) typically had ∼2 water molecules in the first solvation shell, with additional waters in the second and third solvation shells, likely arising from its nonspherical structure. In line with the unique binding of iodide described above and its larger ionic radius (2.20 Å),^69^ the RDF peak for iodide occurred at the second greatest distance. Together, these data indicated the strongest water structuring around chloride, consistent with the hydration enthalpy trend that can be ranked from highest to lowest as follows: chloride > bromide > nitrate > iodide.^69^

**Figure 5:**
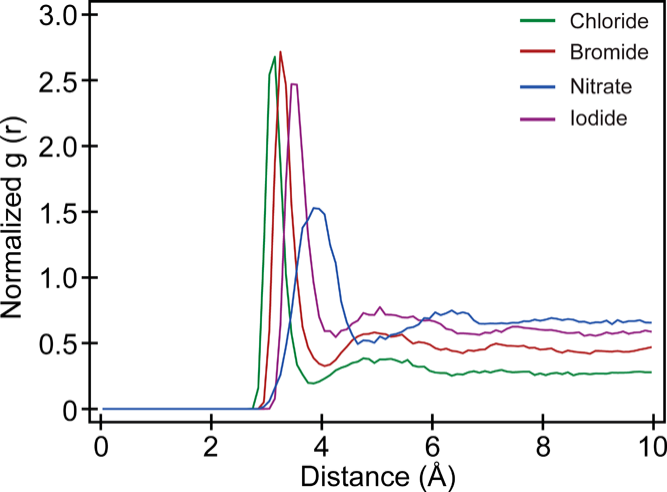
Radial distribution functions computed for the water molecules surrounding each anion. *g*(*r*) is the radius among chloride, bromide, nitrate (nitrogen atom), and iodide with respect to water (oxygen atom).

**Figure 6:**
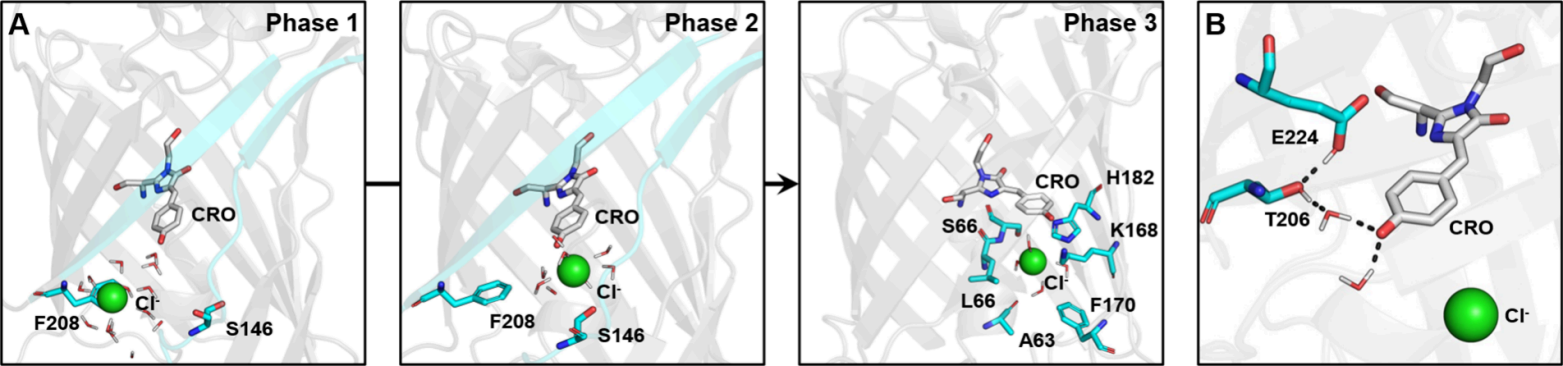
(A) Representative snapshots from the simulated anion entry pathway for chloride binding with the first solvation shell of water in cgreGFP, with (B) a representative snapshot of the potential proton transfer pathway seen upon chloride binding. The overall protein structure is shown with the chromophore (gray), key residues (cyan), and water molecules (red and white). Abbreviations: CRO, chromophore. The opening β7 and β8 strands are labeled and highlighted in cyan.

Once the anion percolated into the protein along with its carrier water molecules, the overall increased hydration in the barrel caused a reorganization of both the chromophore and hydrogen bonding structure (Figure 6). The primary change was a repositioning of water molecules around the phenolate end of the chromophore. Specifically, we observed 1–2 water molecules involved in a hydrogen bonding network above each anion (Figure 6). After repositioning, we posit that the phenolate-bound waters can form possible proton transfer relay networks. This in turn causes a protonation state change for E224 from equivalently protonated on either oxygen to 100% protonated on the oxygen proximal to Thr 206 (Figure 6). We highlight a possible proton transfer pathway via hydrogen bonding with E224 (the equivalent of E222 in GFP) and T206, which have previously been shown to participate in proton transfer pathways in GFP, though we observe others as well.^71–74^ For example, H149 can also alter its protonation state and hydration upon ion binding (Table S2). Such pathways are much more accessible in our simulations upon ion binding, further supporting the hypothesis that cgreGFP may undergo a turn-off fluorescence response via a proton relay network.

### Experimental Validation: Spectroscopy

Building from these *in silico* insights, we purified cgreGFP with a N-terminal polyhistidine tag from *Escherichia coli* (Figures S5 and S6).^42^ In the absence of chloride (∼0.25 mM), cgreGFP had one major absorption band at 486 nm with a shoulder at 460 nm from pH 5 to 8 (Figures S7 and S8).^42^ This feature dramatically decreased in intensity and progressively shifted to 394 nm as a function of lowering the pH to 3 (Figures S7 and S8). Overall, the same behavior was observed in the presence of 100 mM sodium chloride, tracking with the conversion from the phenolate to phenol state of the chromophore (Figures S7 and S8). However, upon closer inspection, the absorption intensity decreased over a narrow regime from pH 4.5 to 5.25, with a clear response to chloride at pH 5 (Figures S7 and S8). Encouraged by this result, we probed the emission profile of cgreGFP across the same pH range.

Excitation of the phenolate state at 470 nm resulted in a peak emission maximum at 500 nm (Figures S7 and S8).^42^ Using these data, we approximated that the chromophore p*K*_a_ shifted from 4.9 to 5.2 with 100 mM sodium chloride, in line with the observed fluorescence quenching (Figures S9 and S10). Intriguingly, excitation of the phenol state at 394 nm also resulted in a peak emission maximum at the exact same wavelength with a gradual shift to 495 nm upon acidification, albeit with lower intensity (Figures S7 and S8). The latter observation was surprising without any prior report but is not unfounded across fluorescent proteins and could be linked to a ground and/or excited state proton transfer process.^37,75,76^ While we speculate this could be the case, the complex nature and lower intensity excitation of the phenol state process was deemed beyond the scope for further investigation in our present study. Thus, we restricted our analysis to the phenolate state.

Knowing that cgreGFP could register the presence of chloride, we next asked if this corresponded to a binding event in which the anion enters into the β-barrel rather than remining on the surface. To probe this, we carried chloride titrations at constant pH of 5 (Figure S11). As can be seen in the absorption spectra in Figure 7A, the phenolate form (λ_abs_ = 485 nm, ε_485_= 40133 ± 269) of the chromophore is favored in the absence of chloride (Figure S12). The addition of chloride triggered a dose-dependent response, shifting the chromophore equilibrium toward the phenol state (λ_abs_ = 394 nm, ε_394_ = 21985 ± 826) (Figure S13). Indeed, this translated to fluorescence quenching with no shift in the emission maximum at 500 nm (Φ_apo_ = 0.85 ± 0.01, Φ_bound_ = 0.72 ± 0.03) (Figure 7B, Figures S14 and S15).^42^ Further fitting of these data indicated that the apparent chloride binding affinity, defined here as *K*_d_, was 25.2 ± 2.0 mM (Figure S16).

**Figure 7:**
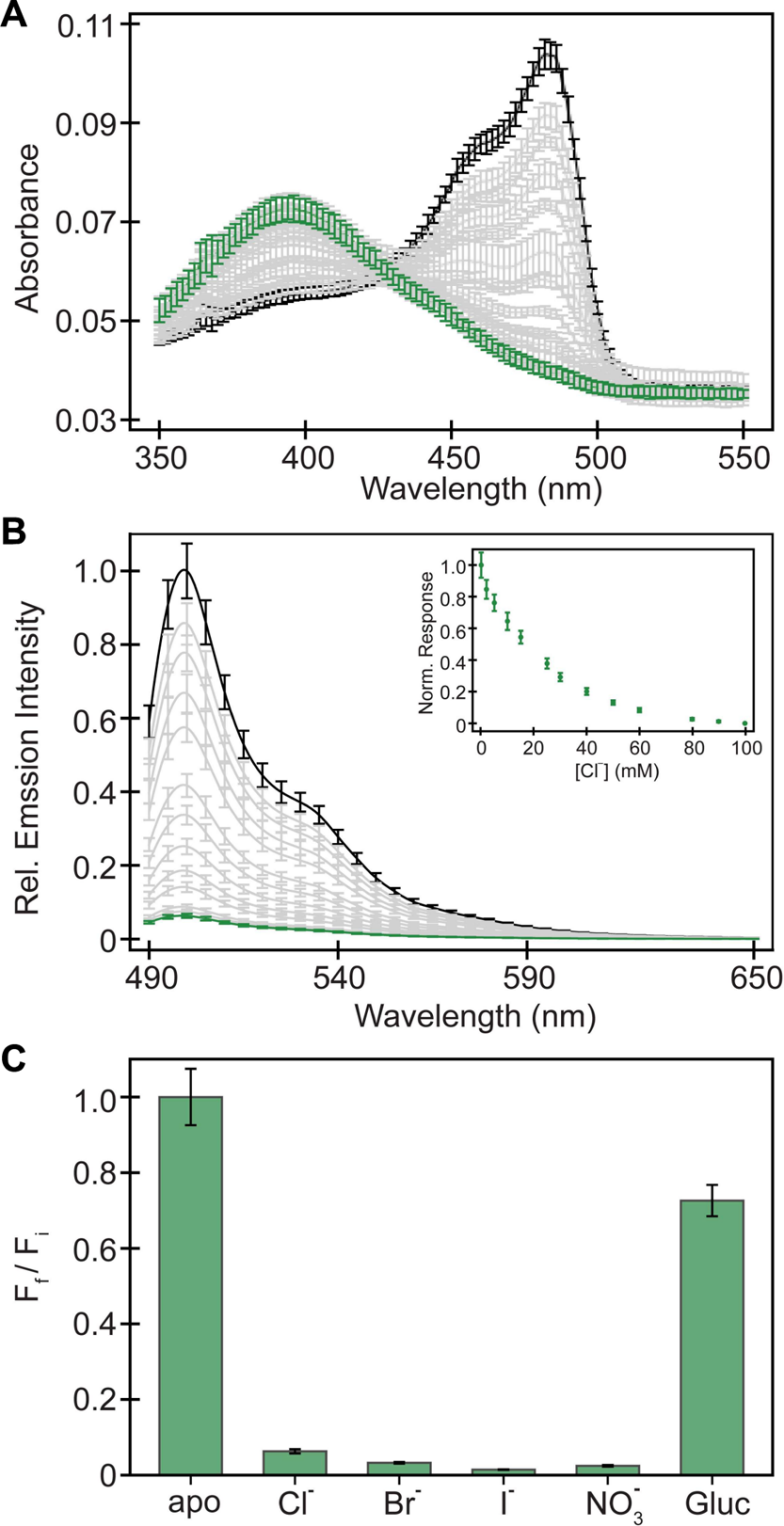
(a) Absorbance and (b) emission responses of cgreGFP to 0 (black) to 100 mM (green) chloride in 20 mM sodium citrate at pH 5 with 0.25 mM sodium chloride. Inset: emission intensity at 500 nm versus the chloride concentration. (c) Emission response of cgreGFP at 500 to 100 mM chloride (Cl^–^), bromide (Br^–^), iodide (I^–^), nitrate (NO_3_^–^), and gluconate (Gluc). For all panels, data from two protein batches are shown as the average with standard deviation of three measurements.

Similar effects were observed with bromide (*K*_d_ = 15.1 ± 1.2 mM), iodide (*K*_d_ = 5.3 ± 0.0 mM), and nitrate (*K*_d_ = 10.7 ± 1.2 mM) but not with gluconate (Figure 7C; Figures S17–S25). Gluconate serves as a nonbinding control and reflects the effect of ionic strength (Figures S24 and S25). Thus, this confirms the presence of a saturable binding cavity in proximity to the chromophore. Despite physical differences between each anion, such promiscuity is not unfounded in anion-sensitive GFPs.^20^ For cgreGFP the highest affinity tracks with size—not shape nor dehydration enthalpy—on the extreme end for iodide as it is the largest.^69^

### Spectroscopic Characterization of MD-Guided cgreGFP Variant

To confirm our predicted binding pathway and cavity, we examined various residues for mutagenesis with both theoretical prediction and experimental validation. While many of the residues differ somewhat in the binding cavity, H149 was positioned on the β7 strand between the observed entry regions and the primary binding cavity for all anions, including iodide and its alternative entry pathway. This suggested a potential role in stabilizing dynamic anion interactions and gating anion entry into the binding cavity. Its protonation state also fluctuated more frequently than other histidine residues in the region, shifting between the deprotonated and singly protonated forms (Table S2). This behavior highlighted a possible role in a proton relay network with the chromophore, and we hypothesized that it was crucial for the turn-off fluorescence response described above.

To test the potential role of H149 in anion recognition, we performed simulations of the alanine mutant (H149A) with chloride as a representative anion. When chloride was initially placed in the binding cavity of H149A, it escaped into the bulk solvent within 20 ns (Figure S26; Supplemental Movie 2). Additionally, chloride did not enter the β-barrel during the free diffusion simulations. This result provided direction for experimental validation.

We pursued evaluating the behavior of the *in silico* generated H149A mutant in purified form. (Tables S3 and S4; Figures S27 and S28). As expected, anion sensitivity was eliminated at pH 5 (Figures S29–S34). However, the chromophore p*K*_a_ shifted from 3.6 to 4.0 with 100 mM sodium chloride (Figures S35–S38). Thus, this points to the possibility of alternative anion entry pathways and/or binding cavities and further reinforces the complex (and exciting) nature of cgreGFP.

## Conclusions

In this study, we used an *in silico* framework to guide and motivate the experimental investigation of cgreGFP as an anion-sensitive fluorescent protein. Constant pH molecular dynamics (CpHMD) simulations revealed multiple potential anion entry pathways and an unprecedented binding cavity located beneath the chromophore. In addition, these simulations enabled us to capture dynamic, atomistic details of how anion binding induces changes in individual residues, the chromophore, and surrounding solvent—all of which cannot be gleaned from static structures alone—to give rise to the photophysical response.

Spectroscopic characterization of purified cgreGFP confirmed that it functions as a turn-off fluorescent biosensor for chloride at acidic pH. In line with YFP-H148Q and related variants, the sensing mechanism is linked to an increase in the chromophore p*K*_a_ upon chloride binding.^20^ Similar spectral changes were observed in the presence of bromide, iodide, and nitrate. To test the *in silico* predictions, we generated the H149A mutant, which eliminated anion sensitivity at pH 5. H149 therefore has a critical role in the anion entry into the β-barrel and in the proton transfer pathway for the photophysical turn-off response.

In the context of known GFP-based biosensors for chloride, cgreGFP is nonconventional: it is a naturally occurring fluorescent protein with no engineered mutations, binds chloride with relatively high affinity in a unique binding cavity, and exhibits excitation ratiometric behavior. Considering these points, cgreGFP is a compelling system for future investigation to not only deepen our photophysical understanding of fluorescent proteins but also as a platform for engineering functional imaging technologies. To close, in lieu of any prior knowledge, our integrated approach proved to be powerful for uncovering the cryptic anion sensing potential of a single fluorescent protein. Looking ahead, we believe that the development of an *in silico* blueprint will enable the discovery, understanding, and designing of this unique function across the GFP family.

## Supplementary Material







## Data Availability

The data in this study is available in the Main Text and Supporting Information. The corresponding authors can be contacted for additional requests. Simulation files including chromophore and nitrate parameters, topology and coordinate files (prmtop, rst7), constant pH configuration (cpin), representative snapshots for all four anions are at Zenodo DOI:10.5281/zenodo.15023056

## Notes

Chloride ion exiting the binding pocket in the simulated H149A mutant (GIF)
Chloride entry visualization movie (MOV)
Table of GFP family sequence alignment; molecular dynamics analysis of ion placement, chromophore rotation, and protein RMSD; table of protonation state changes at pH 5 from constant pH simulations; amino acid and DNA sequences for cgreGFP and H149A mutant, purification data and spectroscopic characterization; tables of mutagenesis primers and PCR conditions; ion-residue distance analysis; list of files from simulations included in the Zenodo database (PDF)

## References

[1] Tsien R. Y. (1998). The Green
Fluorescent Protein. Annu. Rev. Biochem..

[2] Lambert G. G., Depernet H., Gotthard G., Schultz D. T., Navizet I., Lambert T., Adams S. R., Torreblanca-Zanca A., Chu M., Bindels D. S., Levesque V., Moffatt J. N., Salih A., Royant A., Shaner N. C. (2020). Aequorea’s
Secrets Revealed:
New Fluorescent Proteins with Unique Properties for Bioimaging and
Biosensing. PLOS Biol..

[3] Poding L. H., Jägers P., Senen B., Limmon G. V., Herlitze S., Huhn M. (2024). New Observations
of Fluorescent Organisms in the Banda Sea and in
the Red Sea. PLoS One.

[4] Lambert T. J. (2019). FPbase:
A Community-Editable Fluorescent Protein Database. Nat. Methods.

[5] Nasu Y., Shen Y., Kramer L., Campbell R. E. (2021). Structure-
and Mechanism-Guided
Design of Single Fluorescent Protein-Based Biosensors. Nat. Chem. Biol..

[6] Gest A. M. M., Sahan A. Z., Zhong Y., Lin W., Mehta S., Zhang J. (2024). Molecular Spies in Action: Genetically
Encoded Fluorescent Biosensors
Light up Cellular Signals. Chem. Rev..

[7] Cook M. A., Phelps S. M., Tutol J. N., Adams D. A., Dodani S. C. (2025). Illuminating
Anions in Biology with Genetically Encoded Fluorescent Biosensors. Curr. Opin. Chem. Biol..

[8] Mukherjee S., Manna P., Hung S.-T., Vietmeyer F., Friis P., Palmer A. E., Jimenez R. (2022). Directed Evolution
of a Bright Variant of mCherry: Suppression of Nonradiative Decay
by Fluorescence Lifetime Selections. J. Phys.
Chem. B.

[9] Rossano-Tapia M., Brown A. (2022). Quantum Mechanical/Molecular Mechanical
Studies of Photophysical
Properties of Fluorescent Proteins. WIREs Comput.
Mol. Sci..

[10] Mukherjee S., Manna P., Douglas N., Chapagain P. P., Jimenez R. (2023). Conformational Dynamics of mCherry Variants: A Link
between Side-Chain Motions and Fluorescence Brightness. J. Phys. Chem. B.

[11] Illuminating Protein Dynamics: A Review of Computational Methods for Studying Photoactive Proteins. https://arxiv.org/html/2503.21418v1 (accessed 2025–07–07).

[12] Ahmed R. D., Jamieson W. D., Vitsupakorn D., Zitti A., Pawson K. A., Castell O. K., Watson P. D., Jones D. D. (2025). Molecular Dynamics
Guided Identification of a Brighter Variant of Superfolder Green Fluorescent
Protein with Increased Photobleaching Resistance. Commun. Chem..

[13] Ong W. S. Y., Ji K., Pathiranage V., Maydew C., Baek K., Villones R. L. E., Meloni G., Walker A. R., Dodani S. C. (2023). Rational
Design of the β-Bulge Gate in a Green Fluorescent Protein Accelerates
the Kinetics of Sulfate Sensing. Angew. Chem.,
Int. Ed..

[14] Lai C., Yang L., Pathiranage V., Wang R., Subach F. V., Walker A. R., Piatkevich K. D. (2024). Genetically Encoded Green Fluorescent
Sensor for Probing Sulfate Transport Activity of Solute Carrier Family
26 Member A2 (Slc26a2) Protein. Commun. Biol..

[15] Chen C., Pathiranage V., Ong W. S. Y., Dodani S. C., Walker A. R., Fang C. (2025). A Twisted
Chromophore Powers a Turn-on Fluorescent Protein Chloride
Sensor. Proc. Natl. Acad. Sci. U. S. A..

[16] Wachter R. M., James Remington S. (1999). Sensitivity of the Yellow Variant of Green Fluorescent
Protein to Halides and Nitrate. Curr. Biol..

[17] Arosio D., Ratto G. M. (2014). Twenty Years of Fluorescence Imaging of Intracellular
Chloride. Front. Cell. Neurosci..

[18] Zajac M., Chakraborty K., Saha S., Mahadevan V., Infield D. T., Accardi A., Qiu Z., Krishnan Y. (2020). What Biologists
Want from Their Chloride Reporters - a Conversation between Chemists
and Biologists. J. Cell Sci..

[19] Jayaraman S., Haggie P., Wachter R. M., Remington S. J., Verkman A. S. (2000). Mechanism and Cellular Applications
of a Green Fluorescent
Protein-Based Halide Sensor. J. Biol. Chem..

[20] Wachter R. M., Yarbrough D., Kallio K., Remington S. J. (2000). Crystallographic
and Energetic Analysis of Binding of Selected Anions to the Yellow
Variants of Green Fluorescent Protein1. J. Mol.
Biol..

[21] Shinobu A., Agmon N. (2015). The Hole in the Barrel: Water Exchange at the GFP Chromophore. J. Phys. Chem. B.

[22] Lodovichi C., Ratto G. M., Trevelyan A. J., Arosio D. (2022). Genetically Encoded
Sensors for Chloride Concentration. J. Neurosci.
Methods.

[23] Shariati K., Zhang Y., Giubbolini S., Parra R., Liang S., Edwards A., Hejtmancik J. F., Ratto G. M., Arosio D., Ku G. (2022). A Superfolder Green
Fluorescent Protein-Based Biosensor Allows Monitoring
of Chloride in the Endoplasmic Reticulum. ACS
Sens..

[24] Grimley J. S., Li L., Wang W., Wen L., Beese L. S., Hellinga H. W., Augustine G. J. (2013). Visualization
of Synaptic Inhibition with an Optogenetic
Sensor Developed by Cell-Free Protein Engineering Automation. J. Neurosci..

[25] Kuner T., Augustine G. J. (2000). A Genetically
Encoded Ratiometric Indicator for Chloride:
Capturing Chloride Transients in Cultured Hippocampal Neurons. Neuron.

[26] Markova O., Mukhtarov M., Real E., Jacob Y., Bregestovski P. (2008). Genetically
Encoded Chloride Indicator with Improved Sensitivity. J. Neurosci. Methods.

[27] Zhong S., Navaratnam D., Santos-Sacchi J. (2014). A Genetically-Encoded YFP Sensor
with Enhanced Chloride Sensitivity, Photostability and Reduced Ph
Interference Demonstrates Augmented Transmembrane Chloride Movement
by Gerbil Prestin (SLC26a5). PloS One.

[28] Galietta L. J., Haggie P. M., Verkman A. S. (2001). Green Fluorescent
Protein-Based Halide
Indicators with Improved Chloride and Iodide Affinities. FEBS Lett..

[29] Arosio D., Garau G., Ricci F., Marchetti L., Bizzarri R., Nifosì R., Beltram F. (2007). Spectroscopic and Structural
Study of Proton and Halide Ion Cooperative Binding to GFP. Biophys. J..

[30] Mukhtarov M., Liguori L., Waseem T., Rocca F., Buldakova S., Arosio D., Bregestovski P. (2013). Calibration
and Functional Analysis
of Three Genetically Encoded Cl(−)/pH Sensors. Front. Mol. Neurosci..

[31] Paredes J. M., Idilli A. I., Mariotti L., Losi G., Arslanbaeva L. R., Sato S. S., Artoni P., Szczurkowska J., Cancedda L., Ratto G. M., Carmignoto G., Arosio D. (2016). Synchronous Bioimaging of Intracellular pH and Chloride
Based on LSS Fluorescent Protein. ACS Chem.
Biol..

[32] Arosio D., Ricci F., Marchetti L., Gualdani R., Albertazzi L., Beltram F. (2010). Simultaneous Intracellular
Chloride and pH Measurements
Using a GFP-Based Sensor. Nat. Methods.

[33] Raimondo J.
V., Joyce B., Kay L., Schlagheck T., Newey S. E., Srinivas S., Akerman C. J. (2013). A Genetically-Encoded
Chloride and pH Sensor for Dissociating Ion Dynamics in the Nervous
System. Front. Cell. Neurosci..

[34] Tutol J. N., Ong W. S. Y., Phelps S. M., Peng W., Goenawan H., Dodani S. C. (2024). Engineering the
ChlorON Series: Turn-On Fluorescent
Protein Sensors for Imaging Labile Chloride in Living Cells. ACS Cent. Sci..

[35] Yang J., Wang L., Yang F., Luo H., Xu L., Lu J., Zeng S., Zhang Z. (2013). mBeRFP, an
Improved Large Stokes
Shift Red Fluorescent Protein. PLoS One.

[36] Tutol J. N., Kam H. C., Dodani S. C. (2019). Identification
of mNeonGreen as a
pH-Dependent, Turn-On Fluorescent Protein Sensor for Chloride. ChemBioChem..

[37] Chen C., Tutol J. N., Tang L., Zhu L., Ong W. S. Y., Dodani S. C., Fang C. (2021). Excitation Ratiometric
Chloride Sensing
in a Standalone Yellow Fluorescent Protein Is Powered by the Interplay
between Proton Transfer and Conformational Reorganization. Chem. Sci..

[38] Salto R., Giron M. D., Puente-Muñoz V., Vilchez J. D., Espinar-Barranco L., Valverde-Pozo J., Arosio D., Paredes J. M. (2021). New Red-Emitting
Chloride-Sensitive Fluorescent Protein with Biological Uses. ACS Sens..

[39] Peng W., Maydew C. C., Kam H., Lynd J. K., Tutol J. N., Phelps S. M., Abeyrathna S., Meloni G., Dodani S. C. (2022). Discovery
of a Monomeric Green Fluorescent Protein Sensor for Chloride by Structure-Guided
Bioinformatics. Chem. Sci..

[40] Peng W., Tutol J. N., Phelps S. M., Kam H., Lynd J. K., Dodani S. C. (2025). Directed Evolution of a Genetically
Encoded Indicator
for Chloride. ACS Synth. Biol..

[41] Borrell K. L., Cancglin C., Stinger B. L., DeFrates K. G., Caputo G. A., Wu C., Vaden T. D. (2017). An Experimental and Molecular Dynamics Study of Red
Fluorescent Protein mCherry in Novel Aqueous Amino Acid Ionic Liquids. J. Phys. Chem. B.

[42] Markova S. V., Burakova L. P., Frank L. A., Golz S., Korostileva K. A., Vysotski E. S. (2010). Green-Fluorescent
Protein from the Bioluminescent Jellyfish
Clytia Gregaria: cDNA Cloning, Expression, and Characterization of
Novel Recombinant Protein. Photochem. Photobiol.
Sci..

[43] Malikova N. P., Visser N. V., van Hoek A., Skakun V. V., Vysotski E. S., Lee J., Visser A. J. W. G. (2011). Green-Fluorescent Protein from the
Bioluminescent Jellyfish Clytia Gregaria Is an Obligate Dimer and
Does Not Form a Stable Complex with the Ca2+-Discharged Photoprotein
Clytin. Biochemistry.

[44] Williams C. J., Headd J. J., Moriarty N. W., Prisant M. G., Videau L. L., Deis L. N., Verma V., Keedy D. A., Hintze B. J., Chen V. B., Jain S., Lewis S. M., Arendall W. B., Snoeyink J., Adams P. D., Lovell S. C., Richardson J. S., Richardson D. C. (2018). MolProbity:
More and Better Reference Data for Improved
All-Atom Structure Validation. Protein Sci.
Publ. Protein Soc..

[45] Case, D. A. ; Belfon, K. ; Ben-Shalom, I. Y. ; Brozell, S. R. ; Cerutti, D. S. ; Cheatham, T. E., III ; Cruzeiro, V. W. D. ; Darden, T. A. ; Duke, R. E. ; Giambasu, G. ; Gilson, M. K. ; Gohlke, H. ; Goetz, A. W. ; Harris, R. ; Izadi, S. ; Iz-mailov, S. A. ; Kasavajhala, K. ; Kovalenko, A. ; Krasny, R. ; Kurtzman, T. ; Lee, T.S. ; LeGrand, S. ; Li, P. ; Lin, C. ; Liu, J. ; Luchko, T. ; Luo, R. ; Man, V. ; Merz, K. M. ; Miao, Y. ; Mikhailovskii, O. ; Monard, G. ; Nguyen, H. ; Onufriev, A. ; Pan, F. ; Pantano, S. ; Qi, R. ; Roe, D. R. ; Roitberg, A. ; Sagui, C. ; Schott-Verdugo, S. ; Shen, J. ; Simmerling, C. L. ; Skrynnikov, N. R. ; Smith, J. ; Swails, J. ; Walker, R. C. ; Wang, J. ; Wilson, L. ; Wolf, R. M. ; Wu, X. ; Xiong, Y. ; Xue, Y. ; York, D. M. ; Kollman, P. A. AMBER; University of California: San Francisco, 2020.

[46] Case D. A., Cheatham T. E., Darden T., Gohlke H., Luo R., Merz K. M., Onufriev A., Simmerling C., Wang B., Woods R. J. (2005). The AMBER Biomolecular
Simulation
Programs. J. Comput. Chem..

[47] Swails J. M., York D. M., Roitberg A. E. (2014). Constant
pH Replica Exchange Molecular
Dynamics in Explicit Solvent Using Discrete Protonation States: Implementation,
Testing, and Validation. J. Chem. Theory Comput..

[48] Harris J.
A., Liu R., Martins de Oliveira V., Vázquez-Montelongo E. A., Henderson J. A., Shen J. (2022). GPU-Accelerated All-Atom Particle-Mesh
Ewald Continuous Constant pH Molecular Dynamics in Amber. J. Chem. Theory Comput..

[49] Hix M. A., Walker A. R. (2023). AutoParams: An Automated
Web-Based Tool To Generate
Force Field Parameters for Molecular Dynamics Simulations. J. Chem. Inf. Model..

[50] Jorgensen W. L., Chandrasekhar J., Madura J. D., Impey R. W., Klein M. L. (1983). Comparison
of Simple Potential Functions for Simulating Liquid Water. J. Chem. Phys..

[51] Maier J. A., Martinez C., Kasavajhala K., Wickstrom L., Hauser K. E., Simmerling C. (2015). ff14SB: Improving
the Accuracy of
Protein Side Chain and Backbone Parameters from ff99SB. J. Chem. Theory Comput..

[52] Machado M. R., Pantano S. (2020). Split the Charge Difference
in Two! A Rule of Thumb
for Adding Proper Amounts of Ions in MD Simulations. J. Chem. Theory Comput..

[53] Darden T., York D., Pedersen L. (1993). Particle Mesh
Ewald: An N·log­(N)
Method for Ewald Sums in Large Systems. J. Chem.
Phys..

[54] Ryckaert J.-P., Ciccotti G., Berendsen H. J. C. (1977). Numerical Integration of the Cartesian
Equations of Motion of a System with Constraints: Molecular Dynamics
of n-Alkanes. J. Comput. Phys..

[55] Loncharich R. J., Brooks B. R., Pastor R. W. (1992). Langevin
Dynamics of Peptides: The
Frictional Dependence of Isomerization Rates of *N* -acetylalanyl- *N* ′-methylamide. Biopolymers.

[56] Schrödinger, L. L. C. The PyMOL Molecular Graphics System, Version 1.8; Schrodinger, 2015.

[57] Humphrey W., Dalke A., Schulten K. (1996). VMD - Visual
Molecular Dynamics. J. Mol. Graph..

[58] Gasteiger, E. ; Hoogland, C. ; Gattiker, A. ; Duvaud, S. ; Wilkins, M. R. ; Appel, R. D. ; Bairoch, A. Protein Identification and Analysis Tools on the ExPASy Server. In The Proteomics Protocols Handbook; Walker, J. M. , Ed.; Humana Press: Totowa, NJ, 2005; pp 571–607, 10.1385/1-59259-890-0:571.

[59] Shen J., Snook R. D. (1989). Thermal Lens Measurement of Absolute Quantum Yields
Using Quenched Fluorescent Samples as References. Chem. Phys. Lett..

[60] Ewing’s Analytical Instrumentation Handbook, Fourth ed., 4th ed.; Grinberg, N. ; Rodriguez, S. , Eds.; CRC Press: Boca Raton, FL, 2019; 10.1201/9781315118024.

[61] Wood T. I., Barondeau D. P., Hitomi C., Kassmann C. J., Tainer J. A., Getzoff E. D. (2005). Defining the Role of Arginine 96 in Green Fluorescent
Protein Fluorophore Biosynthesis. Biochemistry.

[62] Banerjee S., Schenkelberg C. D., Jordan T. B., Reimertz J. M., Crone E. E., Crone D. E., Bystroff C. (2017). Mispacking and the Fitness Landscape
of the Green Fluorescent Protein Chromophore Milieu. Biochemistry.

[63] Chakravarty S., Ung A. R., Moore B., Shore J., Alshamrani M. (2018). A Comprehensive
Analysis of Anion-Quadrupole Interactions in Protein Structures. Biochemistry.

[64] Kuzniak-Glanowska E., Glanowski M.ł, Kurczab R.ł, Bojarski A. J., Podgajny R. (2022). Mining Anion-Aromatic
Interactions in the Protein Data Bank. Chem.
Sci..

[65] Lucas X., Bauzá A., Frontera A., Quiñonero D. (2016). A Thorough
Anion-π Interaction Study in Biomolecules: On the Importance
of Cooperativity Effects. Chem. Sci..

[66] Nunthaboot N., Tanaka F., Borst J. W., Visser A. J. W. G. (2022). Simultaneous
Analyses of Fluorescence Decay and Anisotropy Decay in Green Fluorescent
Protein Dimer from Jellyfish *Clytia Gregaria*: FRET
and Molecular Dynamics Simulation. J. Photochem.
Photobiol. Chem..

[67] Gonzalez
Somermeyer L., Fleiss A., Mishin A. S., Bozhanova N. G., Igolkina A. A., Meiler J., Alaball Pujol M.-E., Putintseva E. V., Sarkisyan K. S., Kondrashov F. A. (2022). Heterogeneity
of the GFP Fitness Landscape and Data-Driven Protein Design. eLife.

[68] Ivorra-Molla E., Akhuli D., McAndrew M. B. L., Scott W., Kumar L., Palani S., Mishima M., Crow A., Balasubramanian M. K. (2024). A Monomeric
StayGold Fluorescent Protein. Nat. Biotechnol..

[69] Marcus Y. (1994). A Simple Empirical
Model Describing the Thermodynamics of Hydration of Ions of Widely
Varying Charges, Sizes, and Shapes. Biophys.
Chem..

[70] Mizuno H., Mal T. K., Wälchli M., Kikuchi A., Fukano T., Ando R., Jeyakanthan J., Taka J., Shiro Y., Ikura M., Miyawaki A. (2008). Light-Dependent
Regulation of Structural
Flexibility in a Photochromic Fluorescent Protein. Proc. Natl. Acad. Sci. U. S. A..

[71] Donati G., Petrone A., Caruso P., Rega N. (2018). The Mechanism of a
Green Fluorescent Protein Proton Shuttle Unveiled in the Time-Resolved
Frequency Domain by Excited State Ab Initio Dynamics. Chem. Sci..

[72] Petrone A., Cimino P., Donati G., Hratchian H. P., Frisch M. J., Rega N. (2016). On the Driving Force
of the Excited-State
Proton Shuttle in the Green Fluorescent Protein: A Time-Dependent
Density Functional Theory (TD-DFT) Study of the Intrinsic Reaction
Path. J. Chem. Theory Comput..

[73] Di
Donato M., van Wilderen L. J. G.
W., Van Stokkum I. H. M., Stuart T. C., Kennis J. T. M., Hellingwerf K. J., van Grondelle R., Groot M. L. (2011). Proton Transfer Events in GFP. Phys. Chem. Chem. Phys..

[74] Bourne-Worster S., Worth G. A. (2024). Quantum Dynamics
of Excited State Proton Transfer in
Green Fluorescent Protein. J. Chem. Phys..

[75] Chattoraj M., King B. A., Bublitz G. U., Boxer S. G. (1996). Ultra-Fast Excited
State Dynamics in Green Fluorescent Protein: Multiple States and Proton
Transfer. Proc. Natl. Acad. Sci. U. S. A..

[76] Fang C., Frontiera R. R., Tran R., Mathies R. A. (2009). Mapping GFP Structure
Evolution during Proton Transfer with Femtosecond Raman Spectroscopy. Nature.

