# Supplementary material for: Physical Mechanisms of an Unconventional Green Fluorescent Protein Indicator for Chloride
Source: J Phys Chem B. 2026 Mar 5;130(11):3065–75. doi: 10.1021/acs.jpcb.5c08244 (PMC13004581; doi:10.1021/acs.jpcb.5c08244)
Supplement: Supplementary file 3 [file jp5c08244_si_003.pdf]

Supporting Information for

Physical mechanisms of an unconventional green fluorescent protein indicator for chloride

Mfon V. Sunday[a†], Ke Ji[b†], Derik A. Adams[b†], Weicheng Peng[bc], Sheel C. Dodani[b*], Alice R. Walker[a*]

[a]Department of Chemistry, Wayne State University, Detroit, MI 48202
[b]Department of Chemistry and Biochemistry, The University of Texas at Dallas, Richardson, TX 75080
[c]Department of Biological Sciences, The University of Texas at Dallas, Richardson, TX 75080

[†]These authors contributed equally.

Table of Contents

I. Table of GFP family member sequences.
II. Molecular dynamics figures for ion placement, chromophore rotation and protein RMSD.
III. Table of protonation state changes for CpHMD simulations at pH 5.
IV. cgreGFP nucleotide and amino acid sequences.
V. cgreGFP purification and spectroscopy.
VI. Primers for cgreGFP-H149A.
VII. cgreGFP-H149A ion distance data from molecular dynamics simulation.
VIII. Primers for cgreGFP-H149A.
IX. cgreGFP-H149A nucleotide and amino acid sequences.
X. H149A purification and spectroscopy.
XI. List of files from simulations included in Zenodo database.

Figures and Tables

I. Table of GFP family sequences.

Table S1. The 71 members of the GFP family that were aligned and filtered out with at least two of the four binding pocket residues in YFP-H148Q. The members with non-polar amino acids at Q69 and Y203 are highlighted in green.

Entry	UniProt ID	YFP-H148Q binding pocket residues Q69-R88-Q183-Y203
1	tr|A0A6P5AUX2|A0A6P5AUX2_BRABE	YRQL
2	tr|A0A5A4MJQ7|A0A5A4MJQ7_9CNID	SRQL
3	tr|A0A5A4MI65|A0A5A4MI65_9CNID	SRQL
4	tr|A0A5A4MJX1|A0A5A4MJX1_9CNID	SRQL
5	tr|A0A5A4MMK3|A0A5A4MMK3_9CNID	SRQL
6	tr|Q6RYS5|Q6RYS5_9CNID	KRQS
7	tr|Q6RYS6|Q6RYS6_9CNID	KRQS
8	tr|A0A5J6CYN9|A0A5J6CYN9_9CNID	QRQT
9	tr|A0A679KTH4|A0A679KTH4_HUMAN	QRQT
10	tr|A0A6M3ZKY0|A0A6M3ZKY0_BACSU	QRQY
11	tr|U6M5D0|U6M5D0_EIMMA	MRQY
12	tr|U6GSR1|U6GSR1_EIMAC	MRQY
13	tr|A0A125NTU3|A0A125NTU3_HYPSL	QRQY
14	tr|A0A6N9ZS35|A0A6N9ZS35_9HYPH	QRQT
15	tr|A0A6M5E0N3|A0A6M5E0N3_ADE02	QRQT
16	tr|A0A2V2QJP9|A0A2V2QJP9_9ACTN	QRQT
17	tr|A0A2V2QXC3|A0A2V2QXC3_9ACTN	QRQT
18	tr|A0A4Y9T6P2|A0A4Y9T6P2_PSEFL	QRQT
19	tr|A0A076V611|A0A076V611_9VIRU	QRQT
20	tr|A0A0U3A3D8|A0A0U3A3D8_9MONO	QRQT
21	tr|A0A3Q8WKS4|A0A3Q8WKS4_9VIRU	QRQT
22	tr|A0A6G6D467|A0A6G6D467_9HIV1	QRQT
23	tr|B6F2F5|B6F2F5_HE71	QRQT
24	tr|A0A5P9VSM6|A0A5P9VSM6_HRSV	QRQT
25	tr|A0A6L9GAI8|A0A6L9GAI8_9MICC	QRQT
26	tr|Q6YGZ0|Q6YGZ0_9CNID	QRQT
27	tr|A0A6I4B616|A0A6I4B616_9HYPH	QRQT
28	tr|A0A059PIU2|A0A059PIU2_AEQVI	QRQT
29	tr|A0A059PIR9|A0A059PIR9_AEQVI	QRQY
30	tr|A0A059PIQ0|A0A059PIQ0_AEQVI	QRQT
31	tr|Q17106|Q17106_AEQVI	QRQT
32	tr|A0A5J6CYR6|A0A5J6CYR6_AEQVI	QRQT
33	tr|Q17105|Q17105_AEQVI	QRQT
34	tr|I1YZZ9|I1YZZ9_BABBO	QRQT
35	tr|A0A1C3GHU4|A0A1C3GHU4_9ACTN	QRQT
36	tr|A0A1I9LJZ6|A0A1I9LJZ6_9CAUD	QRQT
37	tr|A0A1I9LJ85|A0A1I9LJ85_9CAUD	QRQT
38	tr|A0A1I9LJG8|A0A1I9LJG8_9CAUD	QRQT
39	tr|A0A1I9LJQ6|A0A1I9LJQ6_9CAUD	QRQT
40	tr|A0A2S8V8C2|A0A2S8V8C2_9BACI	QRQT
41	tr|A0A4V4P5F4|A0A4V4P5F4_LEGPN	QRQT
42	tr|A0A4D6FW43|A0A4D6FW43_ECOLI	QRQT
43	tr|Q8GHE4|Q8GHE4_9GAMM	QRQT
44	tr|Q8GHE3|Q8GHE3_AZOVI	QRQT
45	tr|Q8GHE2|Q8GHE2_AZOVI	QRQT
46	tr|W6KDG8|W6KDG8_NICBE	QRQT
47	sp|P42212|GFP_AEQVI	QRQT
48	tr|Q71RY9|Q71RY9_AZOVI	QRQT
49	tr|A0A5J6CYR9|A0A5J6CYR9_AEQVI	QRQT
50	tr|D3TI87|D3TI87_9CNID	QRQT
51	tr|Q8WTC5|Q8WTC5_9CNID	LRQY
52	tr|Q8WTC4|Q8WTC4_9CNID	LRQT
53	tr|Q8WTC7|Q8WTC7_9CNID	LRQC
54	tr|Q8WTC8|Q8WTC8_9CNID	QRQY
55	tr|Q8WTC9|Q8WTC9_9CNID	QRQF
56	tr|Q8WTD0|Q8WTD0_9CNID	QRQL
57	tr|Q8WTC6|Q8WTC6_9CNID	QRQT
58	tr|Q8WP95|Q8WP95_9CNID	QRQT
59	tr|J9PGG2|J9PGG2_9CNID	FRQV
60	tr|D7PM06|D7PM06_CLYGR	FRQV
61	tr|D7PM04|D7PM04_CLYGR	FRQV
62	tr|D7PM10|D7PM10_CLYGR	FRQV
63	tr|D7PM12|D7PM12_CLYGR	FRQV
64	tr|D7PM05|D7PM05_CLYGR	FRQV
65	tr|A0A5J6CYV7|A0A5J6CYV7_9CNID	FRQN
66	tr|A0A5J6CYI7|A0A5J6CYI7_9CNID	LRQN
67	tr|A0A5J6CYT5|A0A5J6CYT5_9CNID	LRQN
68	tr|A0A5J6CYV4|A0A5J6CYV4_AEQVI	LRQN
69	tr|A0A5J6CYK8|A0A5J6CYK8_AEQVI	LRQN
70	tr|G1JSF4|G1JSF4_9CNID	QRQY
71	tr|Q6RYS7|Q6RYS7_9CNID	QRQY

II. Molecular dynamics figures for ion placement, chromophore rotation and protein RMSD.

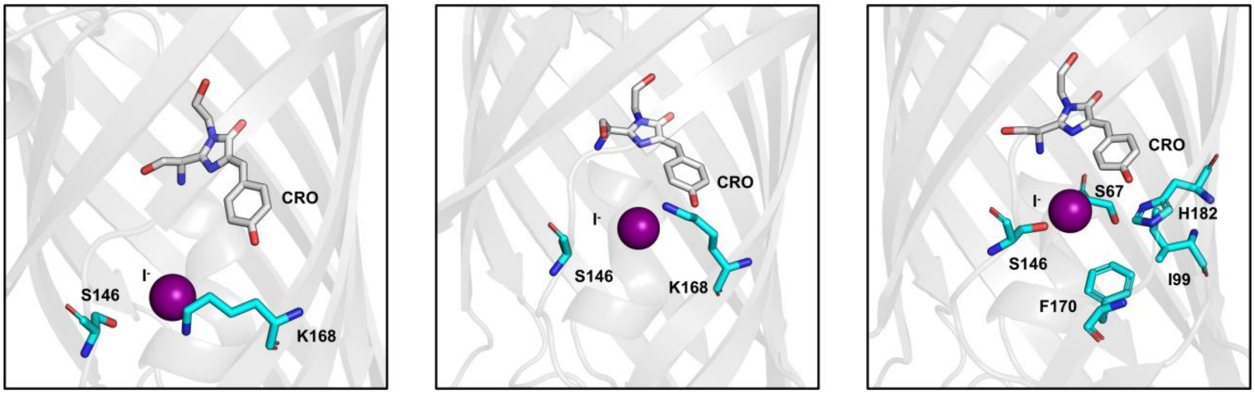

Figure S1. Representative snapshots from the simulated ion entry pathway for iodide in cgreGFP. The overall protein structure is shown with the chromophore (gray) and key residues within 4 Å of the iodide ion (purple). Abbreviation: CRO, chromophore.

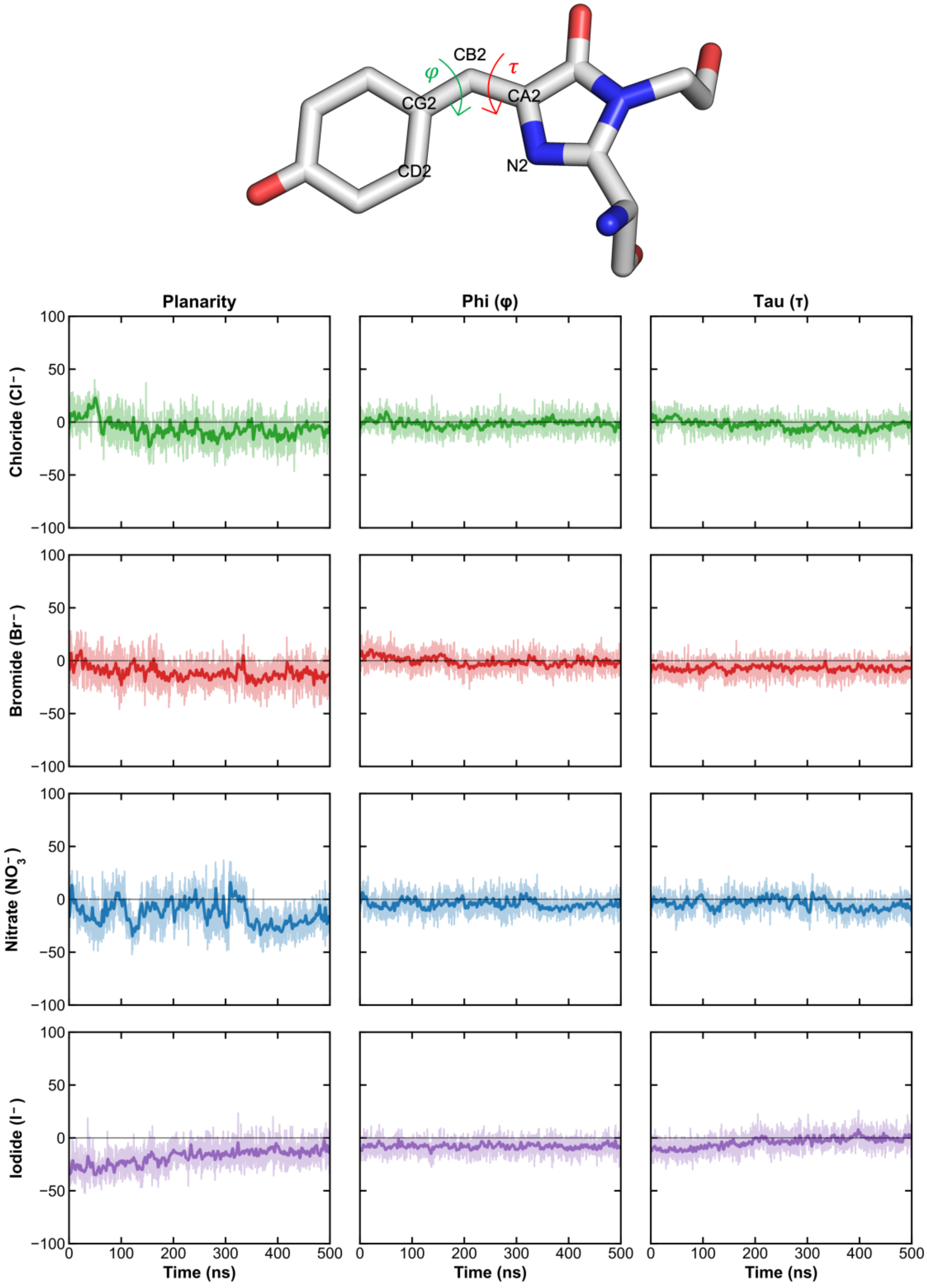

Figure S2. Time evolution of chromophore planarity in the presence of each anion over 500 ns of CpHMD simulations, with a representative structure of the chromophore (gray) and dihedral angles shown with force field atom names. Abbreviation: CpHMD, constant pH molecular dynamics.

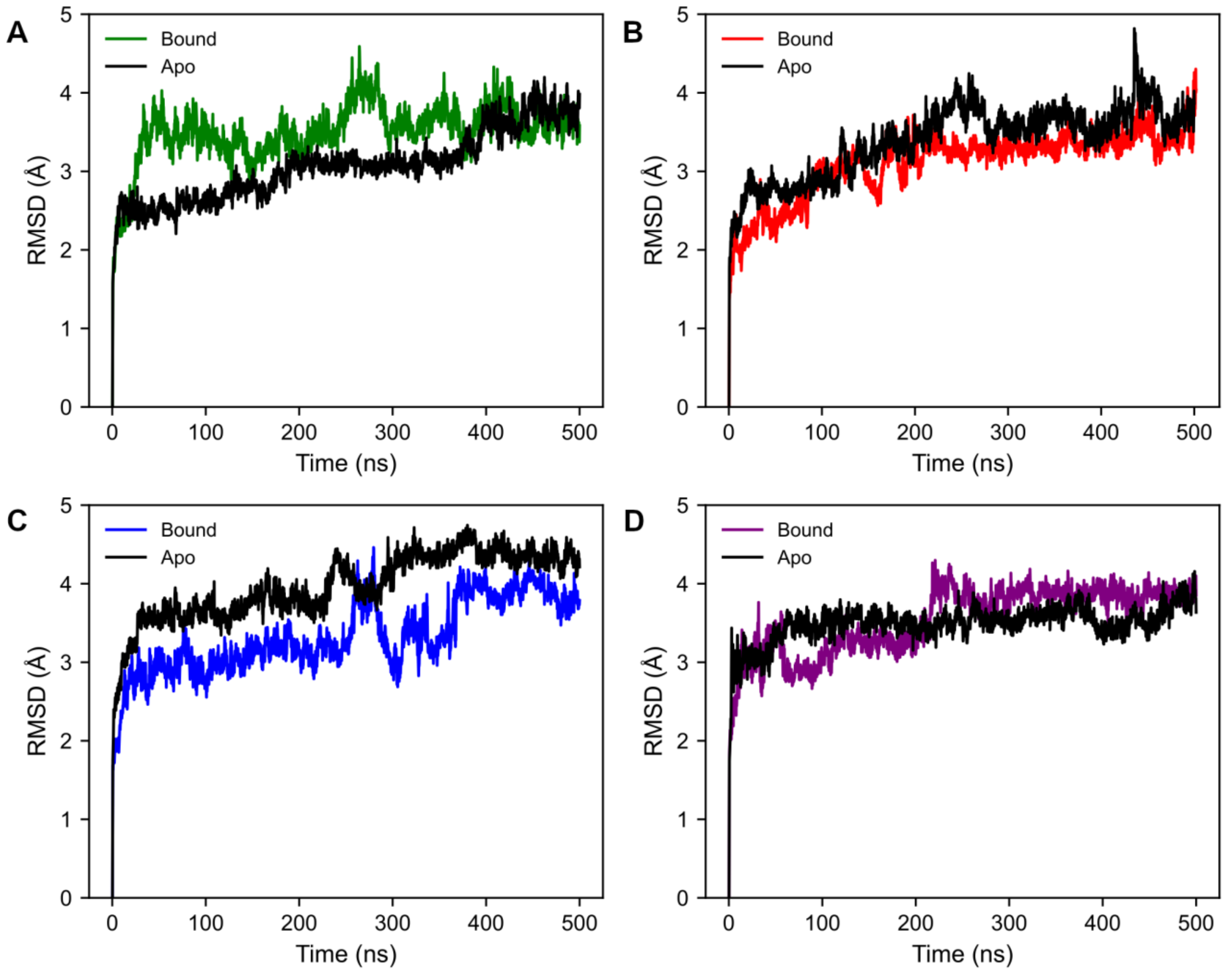

Figure S3. RMSD profiles of the apo (black) and bound (colored) forms of cgreGFP over 500 ns molecular dynamics simulations for (A) chloride, (B) bromide, (C) nitrate, and (D) iodide.

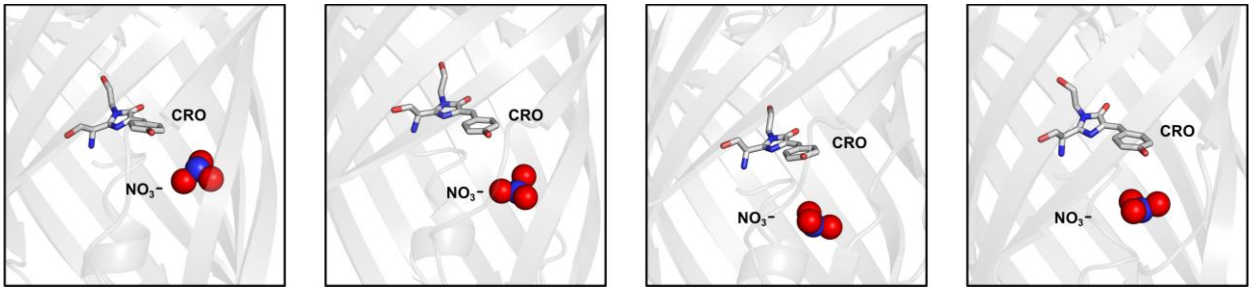

Figure S4. Representative snapshots for metastable nitrate positioning throughout the 500 ns CpHMD simulation. The overall protein structure is shown with the chromophore (gray) and nitrate ion (red and blue). Abbreviations: CpHMD, constant pH molecular dynamics; CRO, chromophore.

III. Table of protonation state changes for CpHMD simulations at pH 5.

Table S2. Fraction of protonation for titratable residues, HIP, GL4, AS4 in the apo and bound forms of cgreGFP with chloride, bromide, nitrate, and iodide at pH 5. Values represent the fraction of time each residue is protonated during the simulation.

Residue	System	Fraction of Protonation	System	Fraction of Protonation
His 149	Chloride Apo	1	Chloride Bound	1
	Bromide Apo	0.794	Bromide Bound	1
	Nitrate Apo	1	Nitrate Bound	0.189
	Iodide Apo	0.998	Iodide Bound	0.726
His 182	Chloride Apo	0.996	Chloride Bound	0.998
	Bromide Apo	0.998	Bromide Bound	0.984
	Nitrate Apo	0.967	Nitrate Bound	0.864
	Iodide Apo	0	Iodide Bound	1
Glu 224	Chloride Apo	1	Chloride Bound	1
	Bromide Apo	1	Bromide Bound	1
	Nitrate Apo	1	Nitrate Bound	1
	Iodide Apo	1	Iodide Bound	1
Asp 96	Chloride Apo	0.011	Chloride Bound	0.195
	Bromide Apo	0.034	Bromide Bound	0.011
	Nitrate Apo	0.014	Nitrate Bound	0.072
	Iodide Apo	0.787	Iodide Bound	1
Glu 165	Chloride Apo	0.002	Chloride Bound	0.566
	Bromide Apo	0.282	Bromide Bound	0.995
	Nitrate Apo	0.003	Nitrate Bound	0.513
	Iodide Apo	0.419	Iodide Bound	0.044
Glu 37	Chloride Apo	0.523	Chloride Bound	0.396
	Bromide Apo	0.507	Bromide Bound	0.47
	Nitrate Apo	0.667	Nitrate Bound	0.626
	Iodide Apo	0.463	Iodide Bound	0.485

IV. cgreGFP nucleotide and amino acid sequences.

ATGGGCAGCAGC**CATCATCATCATCATCAC**AGCAGCGGCGAGAATCTTTATTTTCAGGGC**CATATG**ACCG
CGCTGACCGAAGGCGCGAAGCTGTTCGAGAAAGAAATCCCGTACATTACCGAGCTGGAAGGTGATGTGGA
AGGCATGAAGTTTATCATTAAGGGTGAAGGTACCGGTGATGCGACCACCGGTACCATCAAGGCGAAATAT
ATTTGCACCACCGGTGATCTGCCGGTGCCGTGGGCGACCATCCTGAGCAGCCTGAGCTACGGTGTTTTCT
GCTTTGCGAAATATCCGCGTCACATTGCGGATTTCTTTAAGAGCACCCAGCCGGATGGTTACAGCCAAGA
CCGTATCATTAGCTTCGATAACGACGGCCAGTATGACGTGAAGGCGAAAGTTACCTACGAAAACGGTACC
CTGTATAACCGTGTGACCGTTAAGGGTACCGGCTTTAAAAGCAACGGTAACATCCTGGGCATGCGTGTGC
TGTACCACAGCCCGCCGCACGCGGTTTATATCCTGCCGGATCGTAAGAACGGTGGCATGAAAATTGAGTA
CAACAAGGCGTTCGACGTTATGGGTGGCGGTCACCAGATGGCGCGTCACGCGCAATTTAACAAACCGCTG
GGCGCGTGGGAGGAAGATTACCCGCTGTATCACCACCTGACCGTGTGGACCAGCTTCGGTAAAGATCCGG
ACGATGACGAGACCGACCACCTGACCATCGTGGAAGTTATTAAGGCGGTTGACCTGGAGACCTATCGT**TA**
A

MGSS**HHHHHH**SSGENLYFQG**HM**TALTEGAKLFEKEIPYITELEGDVEGMKFIIKGEGTGDATTGTIKAKY
ICTTGDLPVPWATILSSLSYGVFCFAKYPRHIADFFKSTQPDGYSQDRIISFDNDGQYDVKAKVTYENGT
LYNRVTVKGTGFKSNGNILGMRVLYHSPPHAVYILPDRKNGGMKIEYNKAFDVMGGGHQMARHAQFNKPL
GAWEEDYPLYHHLTVWTSFGKDPDDDETDHLTIVEVIKAVDLETYR*****

Figure S5. Nucleotide (top) and amino acid (bottom) sequences of cgreGFP. The N-terminal polyhistidine tag, NdeI restriction enzyme site, and stop codon (*) are shown in purple, gray, and red colored font, respectively.

V. cgreGFP purification and spectroscopy.

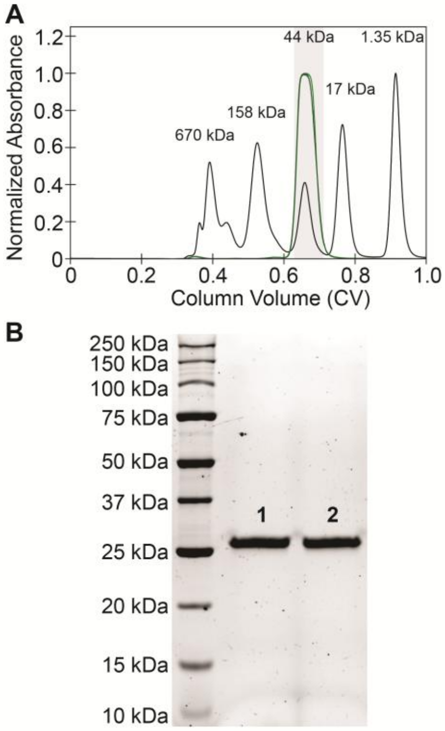

Figure S6. (A) Size exclusion chromatography (SEC) chromatogram for the first batch of cgreGFP (dark green), second batch of cgreGFP (green), and gel filtration standard (black) in 20 mM Tris buffer at pH 7.5 with 150 mM NaCl. All spectra are normalized to the absorption at 280 nm. The molecular weight of each protein standard is labeled on the corresponding peaks. For each cgreGFP batch, the fraction collected for further analysis is denoted in the gray region. (B) Stain-free SDS-PAGE for purified batches of cgreGFP. From left to right: protein ladder, first protein batch (1), and second protein batch (2). The theoretical molecular weight is ~28.4 kDa.

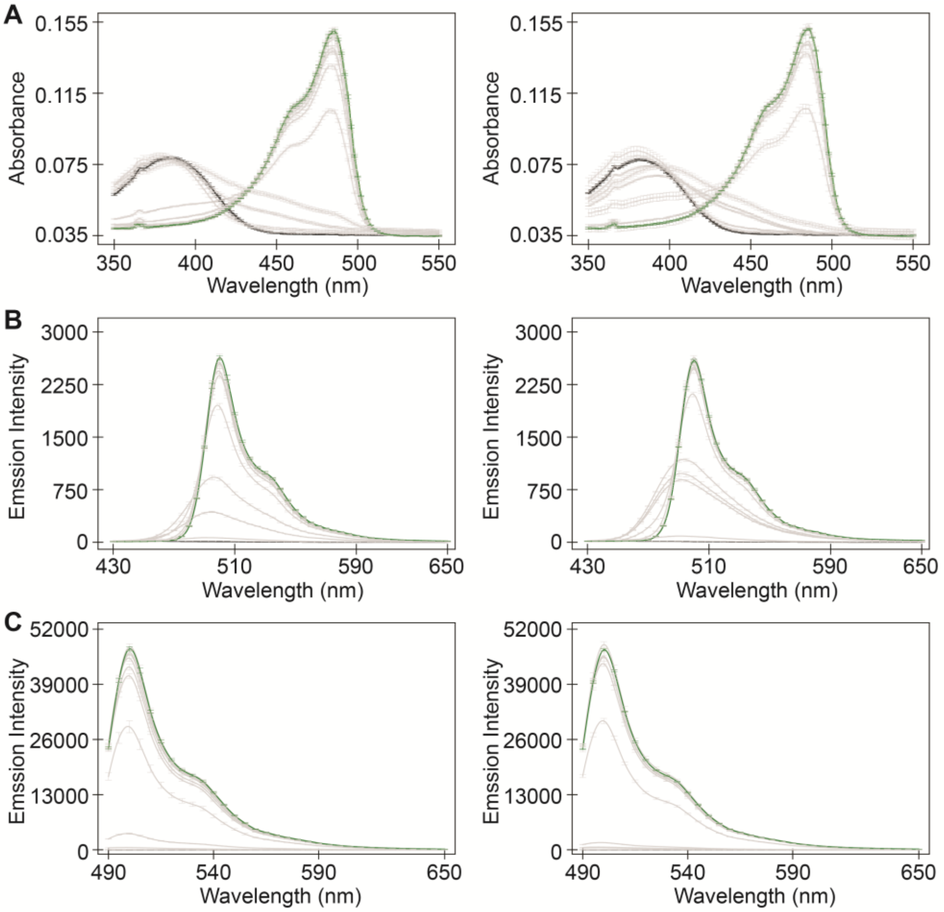

Figure S7. (A) Absorbance and emission spectra of cgreGFP with excitation provided at (B) 394 nm and (C) 470 nm in the absence (left panels) and presence (right panels) of 100 mM sodium chloride from pH 3 (black) to pH 8 (green). Data is shown for the first protein batch as the average of three technical measurements with the standard deviation.

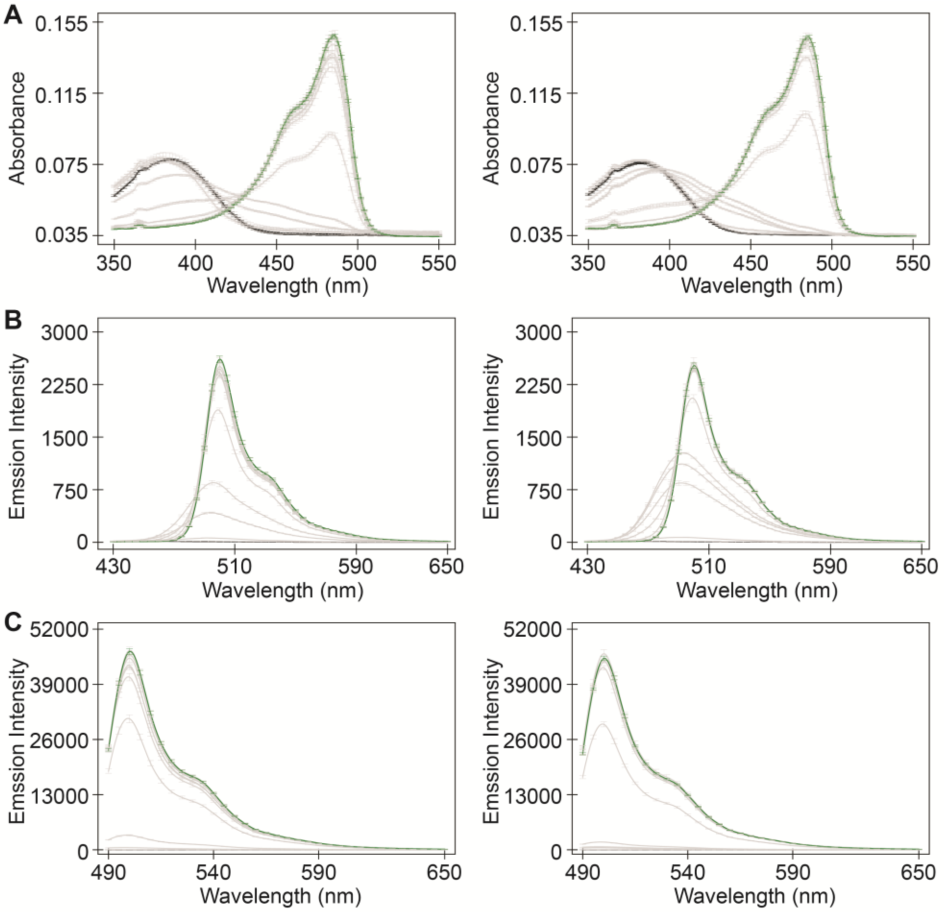

Figure S8. (A) Absorbance and emission spectra of cgreGFP with excitation provided at (B) 394 nm and (C) 470 nm in the absence (left panels) and presence (right panels) of 100 mM sodium chloride from pH 3 (black) to pH 8 (green). Data is shown for the second protein batch as the average of three technical measurements with the standard deviation.

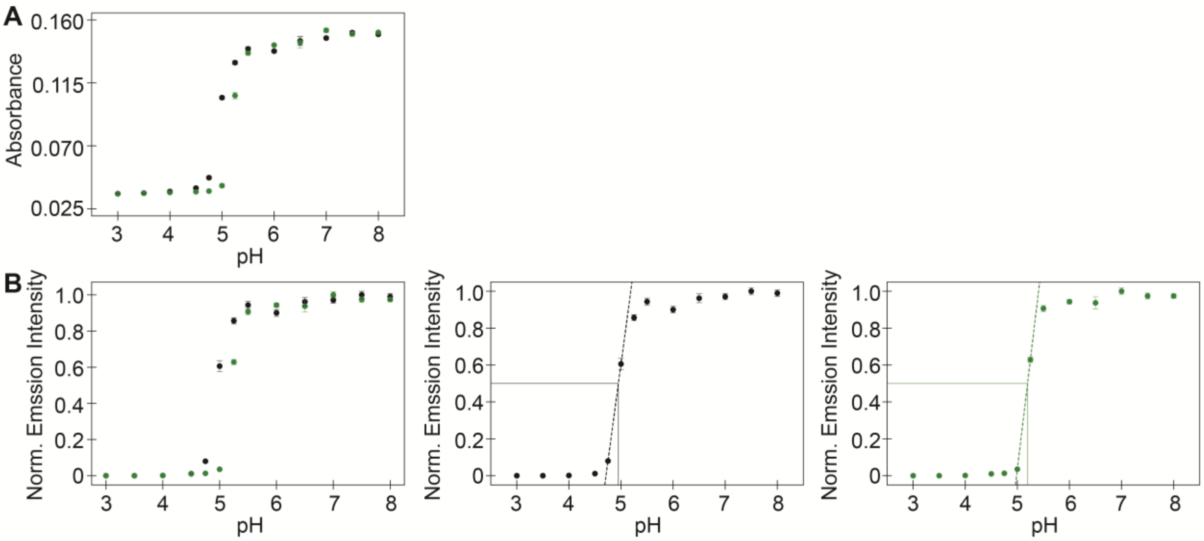

Figure S9. (A) The maximum absorbance intensity (λ_{abs} = 486 nm) plotted versus the pH in the absence (black) and presence of 100 mM sodium chloride (green). (B) The normalized emission response (λ_{ex} = 470 nm, λ_{em} = 500 nm) in the absence (black) and presence of 100 mM sodium chloride (green) plotted versus the pH (left panel). The estimation of the pK_a for cgreGFP in the absence (4.95, middle panel) and presence of 100 mM sodium chloride (5.20, right panel) at which 50% of the fluorescence signal is retained. Data is shown for the first protein batch.

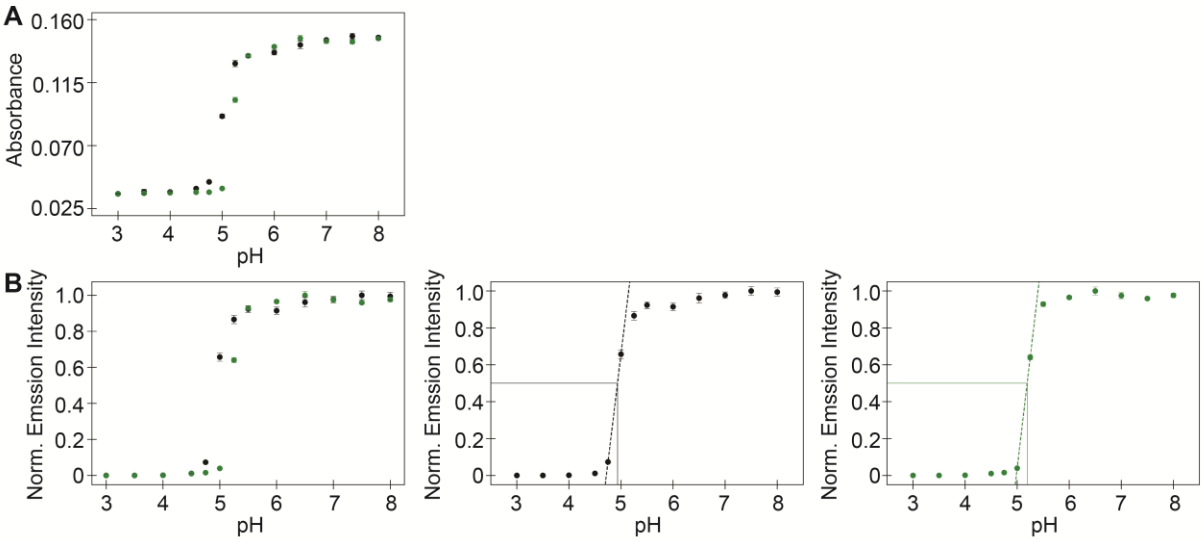

Figure S10. (A) The maximum absorbance intensity (λ_{abs} = 486 nm) plotted versus the pH in the absence (black) and presence of 100 mM sodium chloride (green). (B) The normalized emission response (λ_{ex} = 470 nm, λ_{em} = 500 nm) in the absence (black) and presence of 100 mM sodium chloride (green) plotted versus the pH (left panel). The estimation of the pK_a for cgreGFP in the absence (4.93, middle panel) and presence of 100 mM sodium chloride (5.19, right panel) at which 50% of the fluorescence signal is retained. Data is shown for the second protein batch.

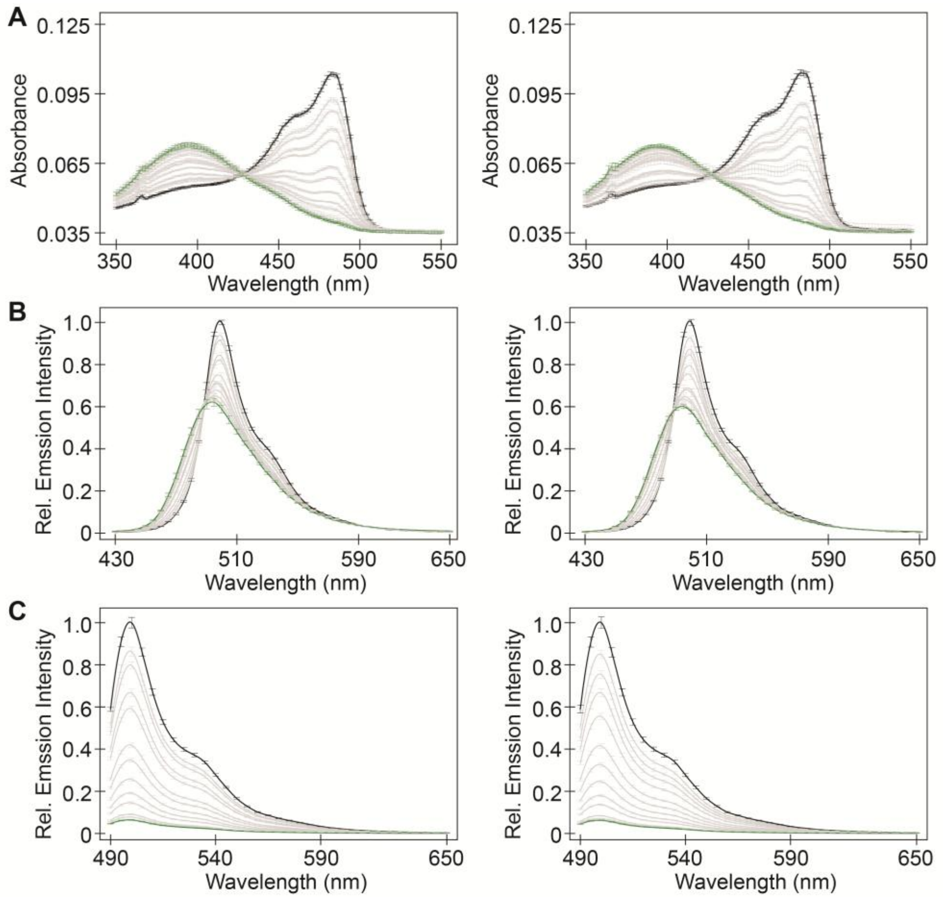

Figure S11. (A) Absorbance and normalized emission spectra of cgreGFP with excitation provided at (B) 394 nm and (C) 470 nm with 0 (black), 2, 5, 10, 15, 25, 30, 40, 50, 60, 80, 90 mM (gray), and 100 mM (green) sodium chloride in 20 mM sodium citrate at pH 5. Data is shown for the first (left panels) and second (right panels) protein batch as the average of three technical measurements with the standard deviation.

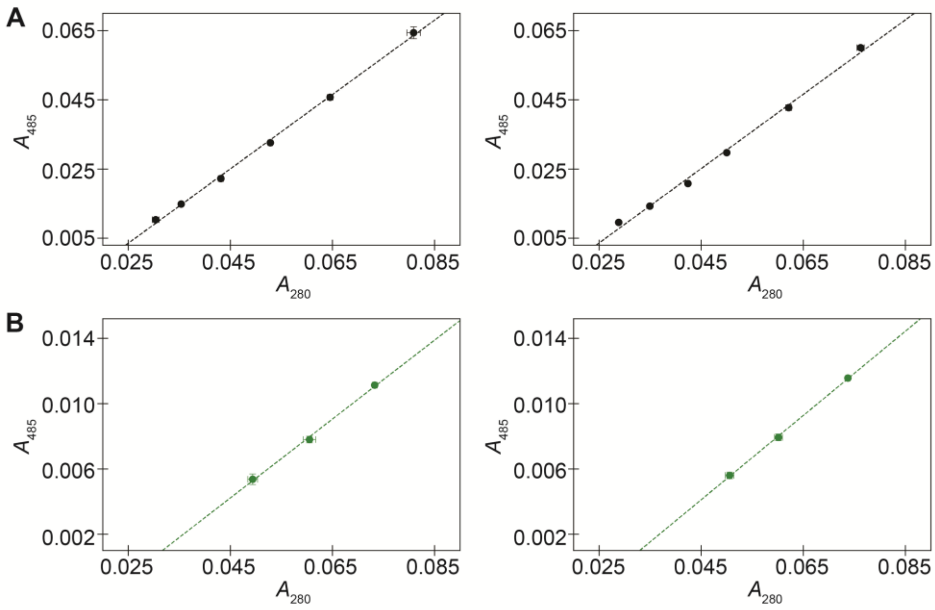

Figure S12. The corrected absorbance intensity at 485 nm (A_{485}) versus the corrected absorbance intensity at 280 nm (A_{280}) ($R^2 > 0.99$) to determine the molar extinction coefficient of cgreGFP at 485 nm in the (A) absence and (B) presence of 100 mM sodium chloride. Data is shown for the first (left panels) and second (right panels) protein batch as the average of three technical measurements with the standard deviation.

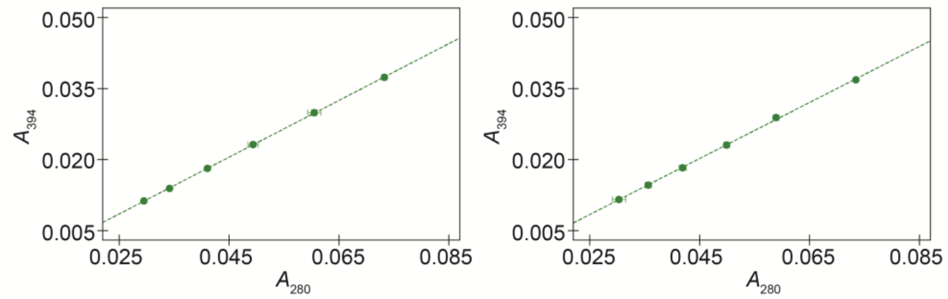

Figure S13. The corrected absorbance intensity at 394 nm (A_{394}) versus the corrected absorbance intensity at 280 nm (A_{280}) ($R^2 > 0.99$) to determine the molar extinction coefficient of cgreGFP at 394 nm in the presence of 100 mM sodium chloride. Data is shown for the first (left panel) and second (right panel) protein batch as the average of three technical measurements with the standard deviation.

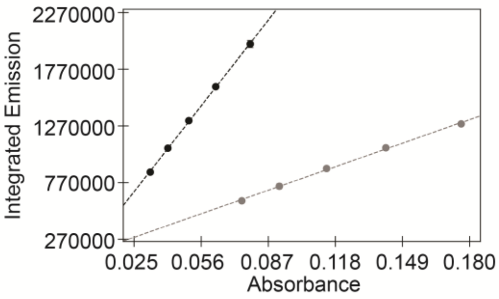

Figure S14. The fluorescence quantum yield standard curves for fluorescein in 0.1 M NaOH (black) and coumarin 135 in 50% ethanol (gray). The integrated emission of each sample is plotted versus the corrected absorbance intensity at 488 nm for fluorescein and 425 nm for coumarin 135 ($R^2 > 0.99$). Data at each concentration was measured in triplicate and is plotted as the average with the standard deviation.

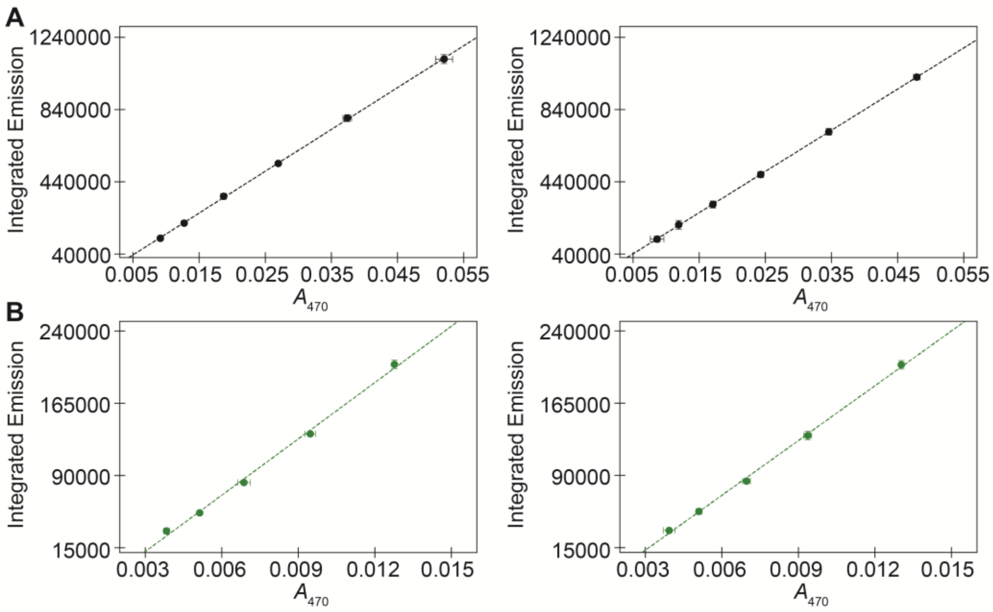

Figure S15. The integrated emission of cgreGFP versus the corrected absorbance intensity at 470 nm ($R^2 > 0.99$) in the (A) absence (B) presence of 100 mM sodium chloride to determine the quantum yield. Data is shown for the first (left panels) and second (right panels) protein batch as the average of three technical measurements with the standard deviation.

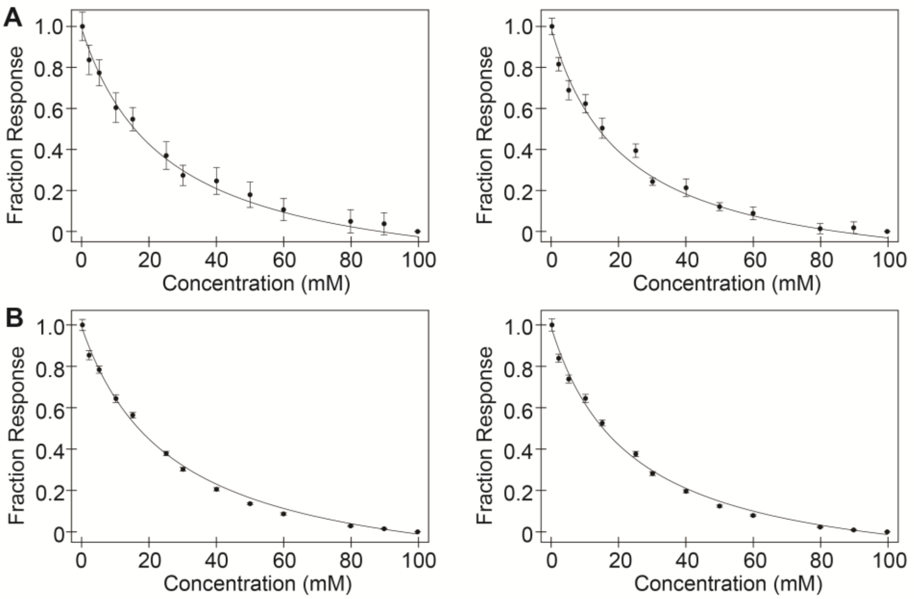

Figure S16. The normalized emission response of cgreGFP at 500 nm with excitation provided at (A) 394 nm and (B) 470 nm fitted to determine the apparent dissociation constant (K_d) for sodium chloride. Data is shown for the first (left panels) and second (right panels) protein batch as the average of three technical measurements with the standard deviation. Note: For panel A, the K_d = 25 ± 1.3 mM.

.

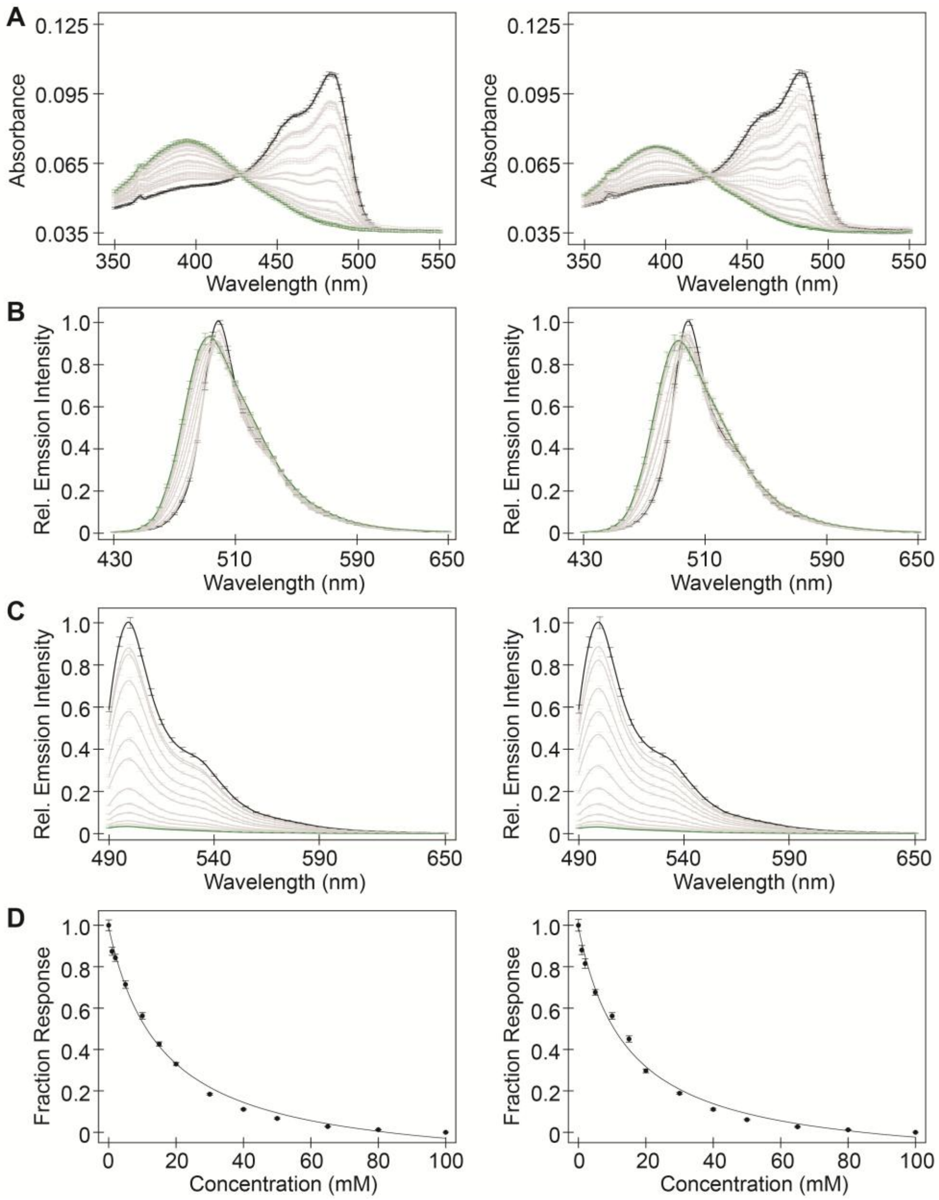

Figure S17. (A) Absorbance and normalized emission spectra of cgreGFP with excitation provided at (B) 394 nm and (C) 470 nm with 0 (black), 1, 2, 5, 10, 15, 20, 30, 40, 50, 65, 80 mM (gray), and 100 mM (green) sodium bromide in 20 mM sodium citrate at pH 5. (D) The emission response in panel (C) fitted to determine the K_d for sodium bromide. Data is shown for the first (left panels) and second (right panels) protein batch as the average of three technical measurements with the standard deviation.

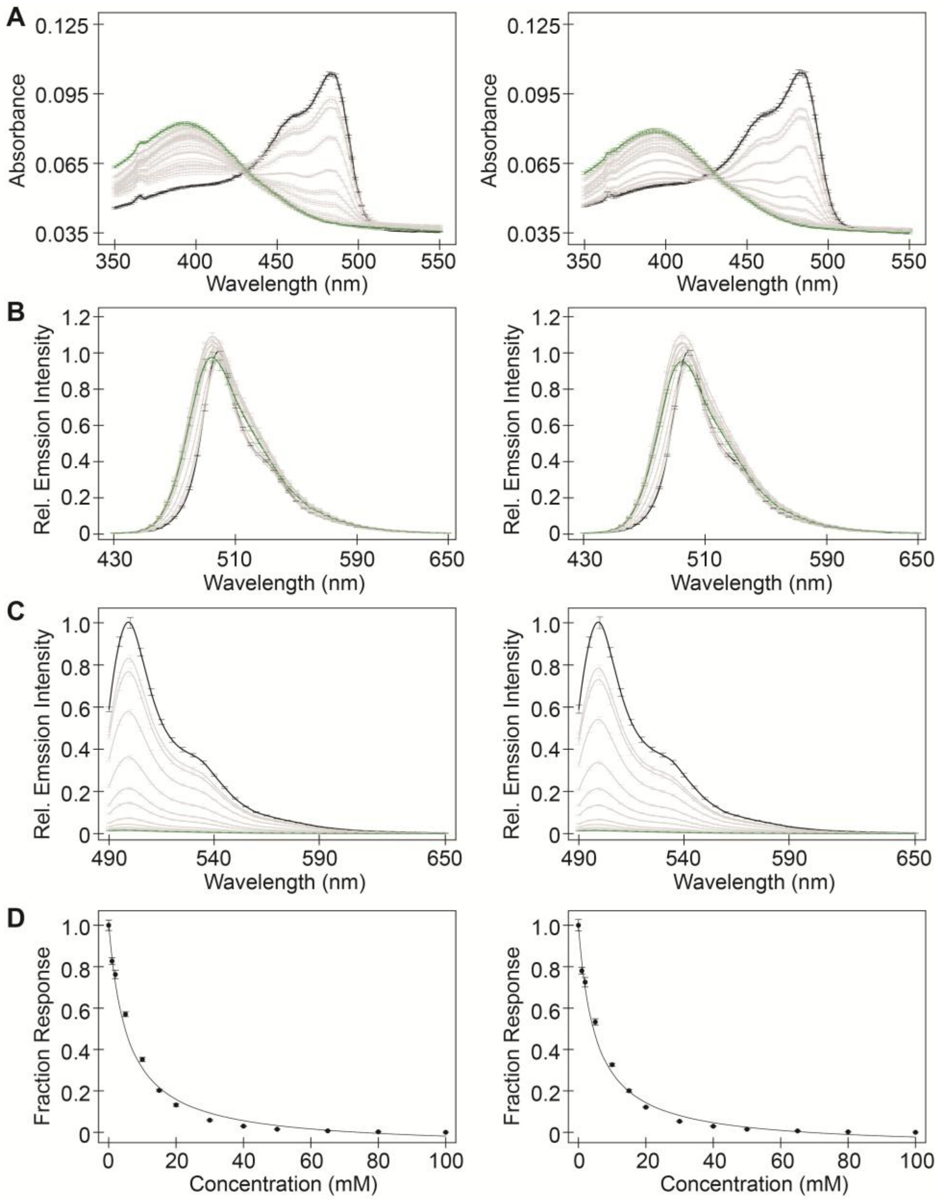

Figure S18. (A) Absorbance and normalized emission spectra of cgreGFP with excitation provided at (B) 394 nm and (C) 470 nm with 0 (black), 1, 2, 5, 10, 15, 20, 30, 40, 50, 65, 80 mM (gray), and 100 mM (green) sodium iodide in 20 mM sodium citrate at pH 5. (D) The emission response from panel (C) fitted to determine the K_d for sodium iodide. Data is shown for the first (left panels) and second (right panels) protein batches as the average of three technical measurements with the standard deviation.

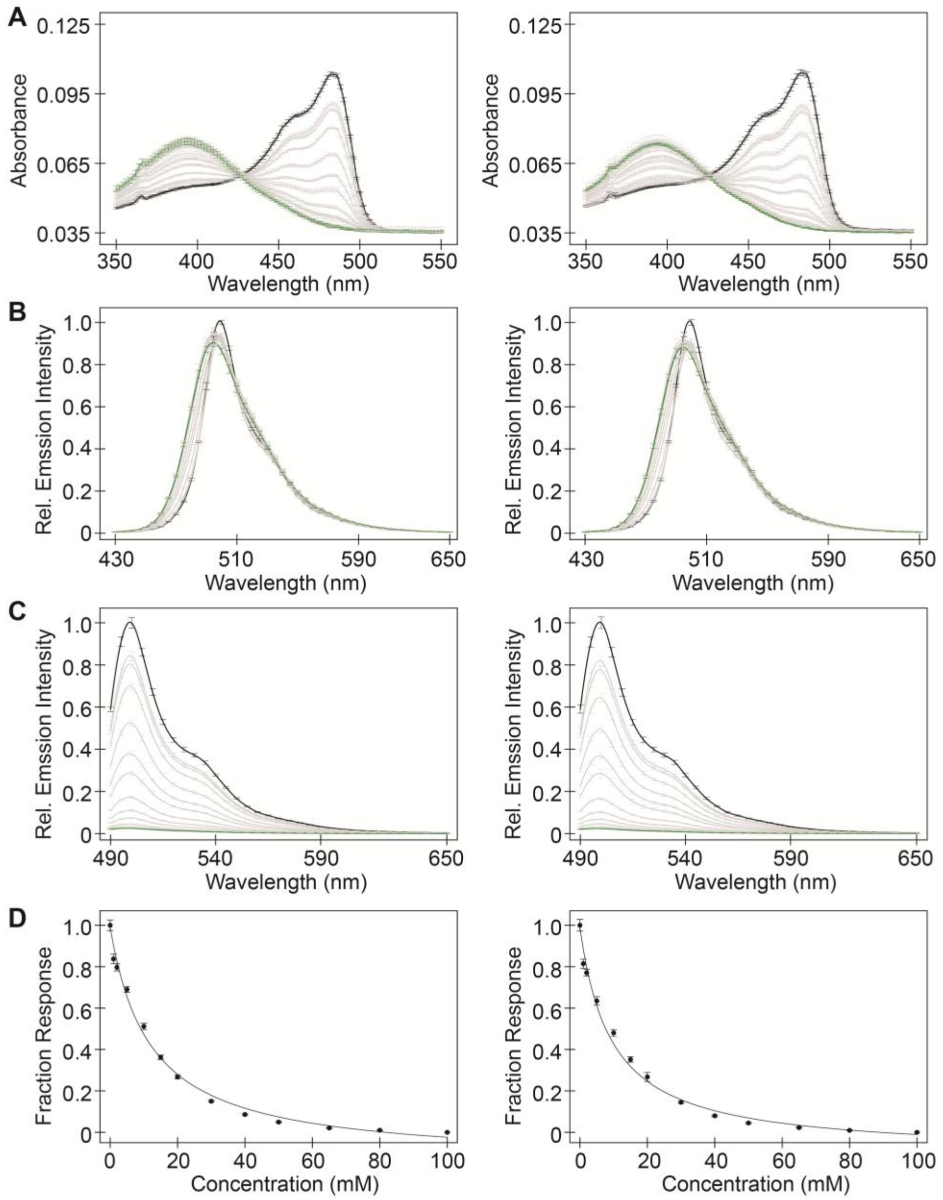

Figure S19. (A) Absorbance and normalized emission spectra of *cgre*GFP with excitation provided at (B) 394 nm and (C) 470 nm with 0 (black), 1, 2, 5, 10, 15, 20, 30, 40, 50, 65, 80 mM (gray), and 100 mM (green) sodium nitrate in 20 mM sodium citrate at pH 5. (D) The emission response from panel (C) fitted to determine the K_d for sodium nitrate. Data is shown for the first (left panels) and second (right panels) protein batches as the average of three technical measurements with the standard deviation.

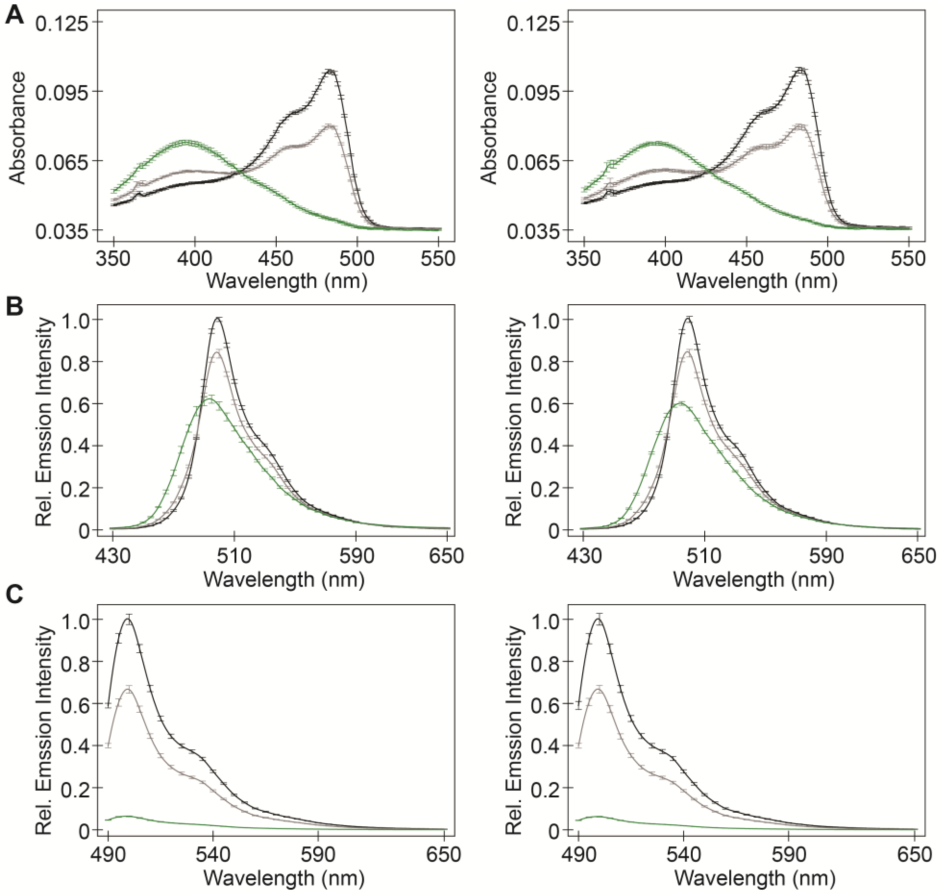

Figure S20. (A) Absorbance and normalized emission spectra of cgreGFP with excitation provided at (B) 394 nm and (C) 470 nm with 0 (black), 10 mM (gray), and 100 mM (green) sodium chloride in 20 mM sodium citrate at pH 5. Data is shown for the first (left panels) and second (right panels) protein batches as the average of three technical measurements with the standard deviation.

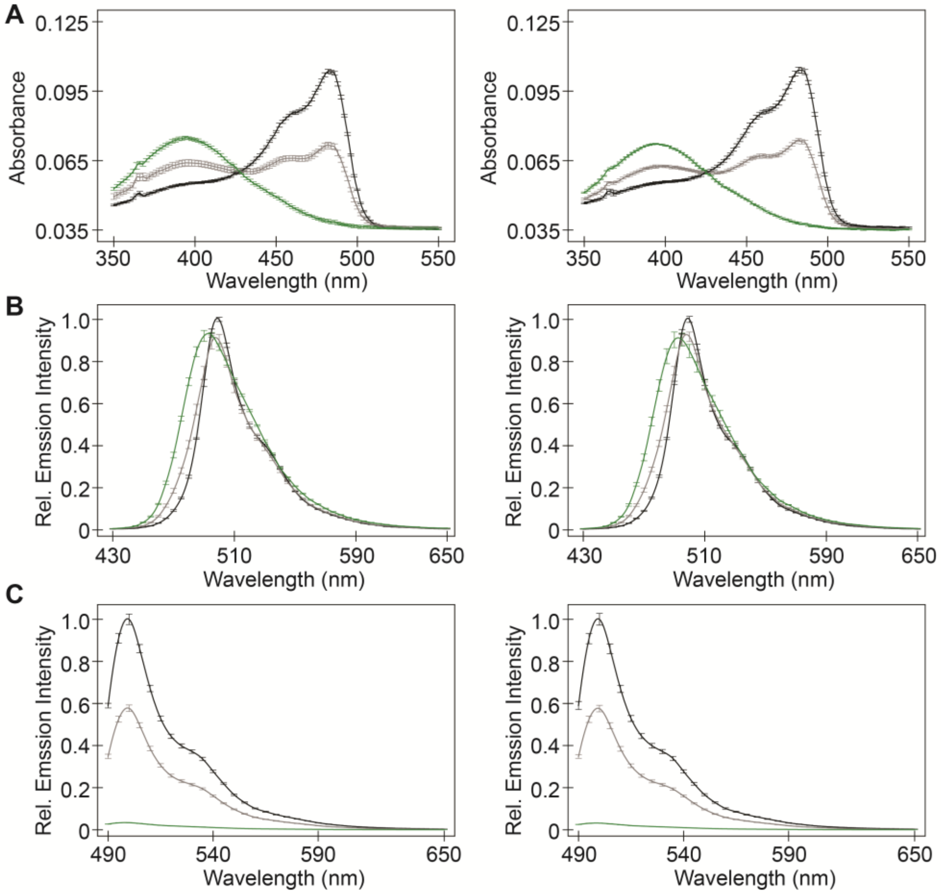

Figure S21. (A) Absorbance and normalized emission spectra of cgreGFP with excitation provided at (B) 394 nm and (C) 470 nm with 0 (black), 10 mM (gray), and 100 mM (green) sodium bromide in 20 mM sodium citrate at pH 5. Data is shown for the first (left panels) and second (right panels) protein batch as the average of three technical measurements with the standard deviation.

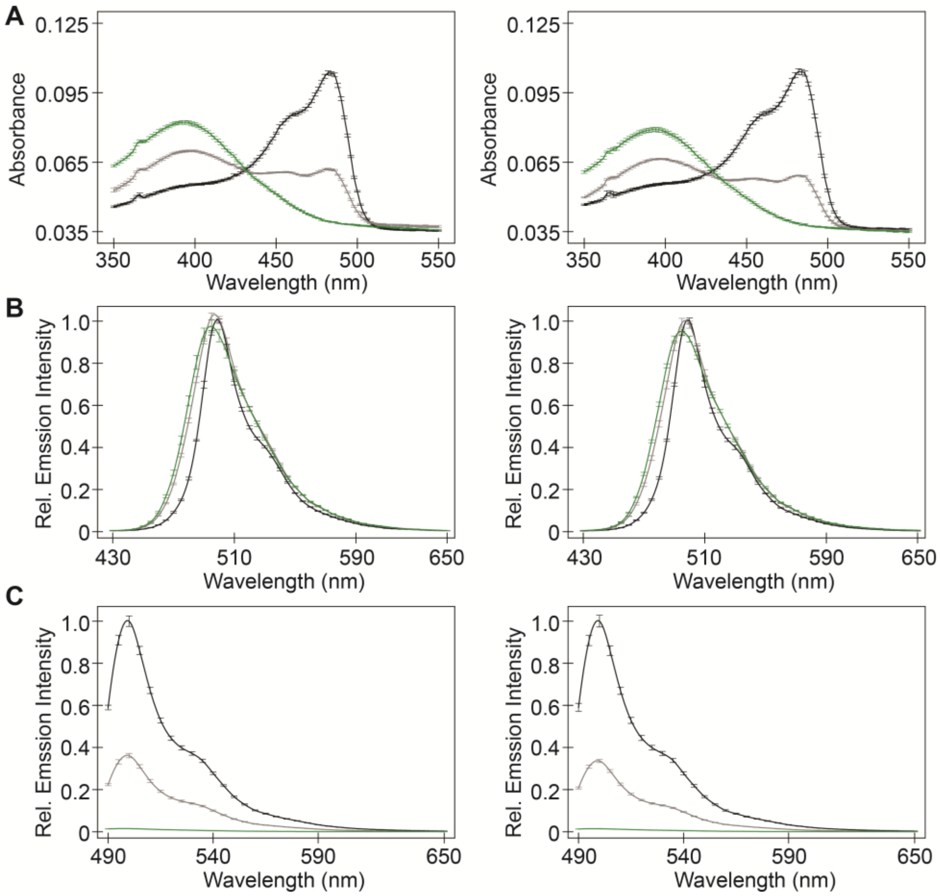

Figure S22. (A) Absorbance and normalized emission spectra of cgreGFP with excitation provided at (B) 394 nm and (C) 470 nm with 0 (black), 10 mM (gray), and 100 mM (green) sodium iodide in 20 mM sodium citrate at pH 5. Data is shown for the first (left panels) and second (right panels) protein batch as the average of three technical measurements with the standard deviation.

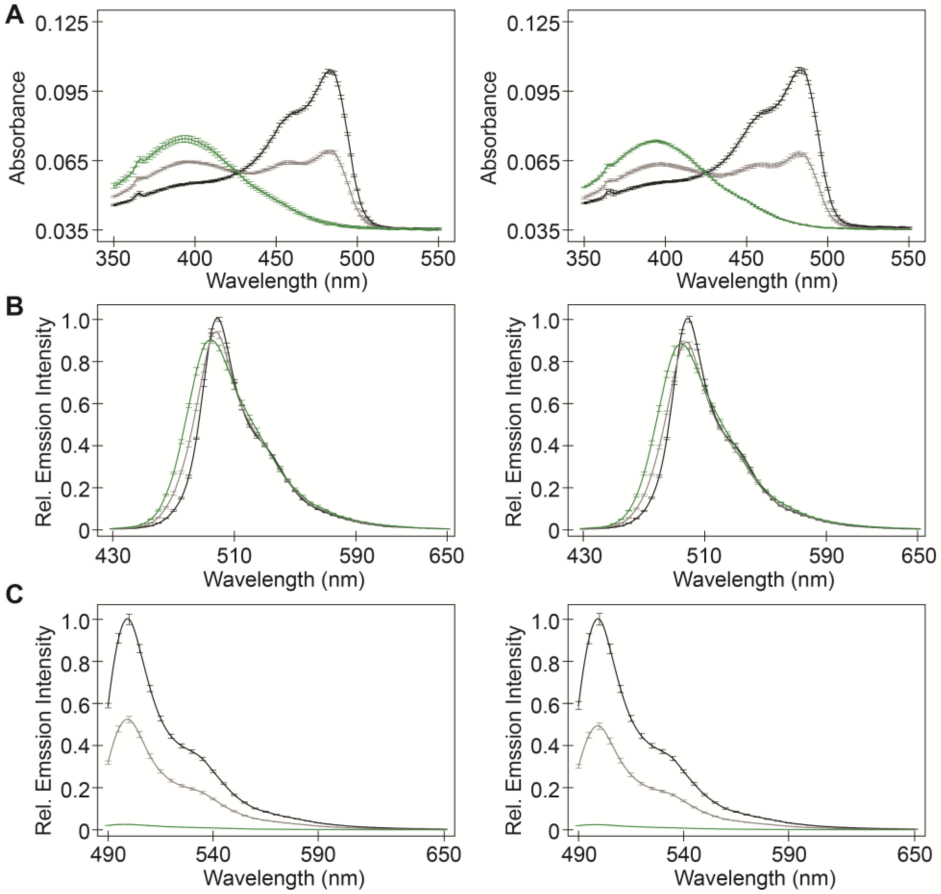

Figure S23. (A) Absorption and normalized emission spectra of cgreGFP with excitation provided at (B) 394 nm and (C) 470 nm with 0 (black), 10 mM (gray), and 100 mM (green) sodium nitrate in 20 mM sodium citrate at pH 5. Data is shown for the first (left panels) and second (right panels) protein batch as the average of three technical measurements with the standard deviation.

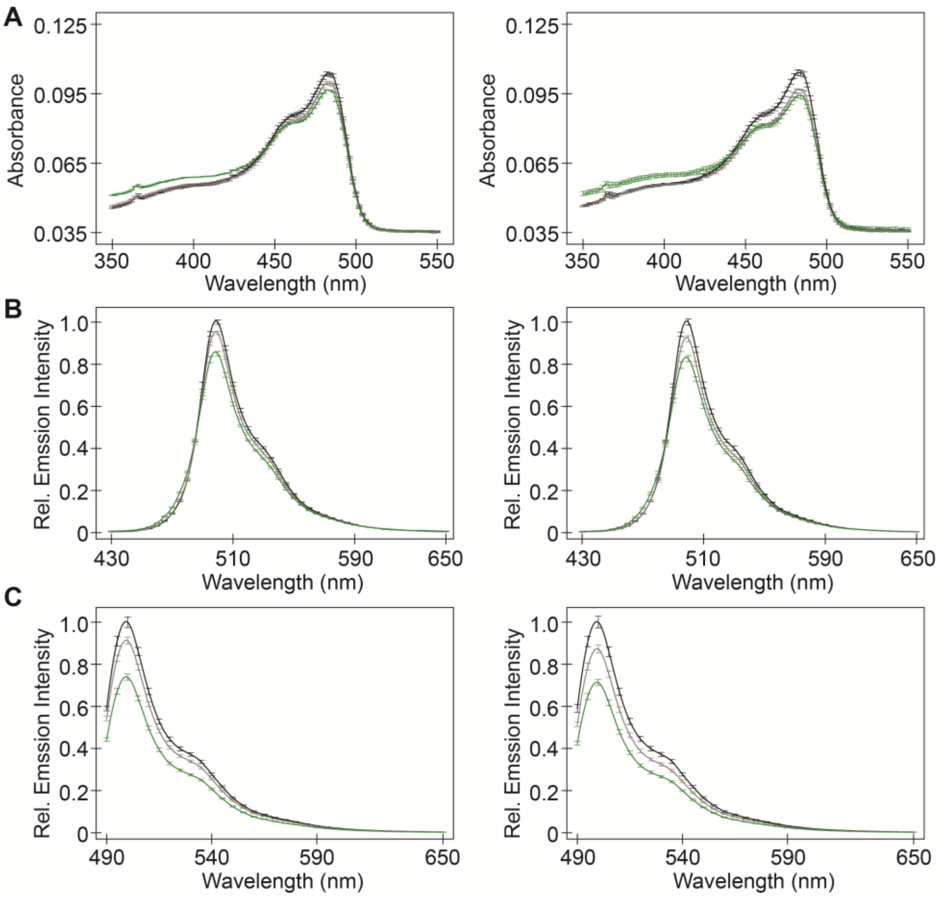

Figure S24. (A) Absorption and normalized emission spectra of cgreGFP with excitation provided at (B) 394 nm and (C) 470 nm with 0 (black), 10 mM (gray), and 100 mM (green) sodium gluconate in 20 mM sodium citrate at pH 5. Data is shown for the first (left panels) and second (right panels) protein batch as the average of three technical measurements with the standard deviation.

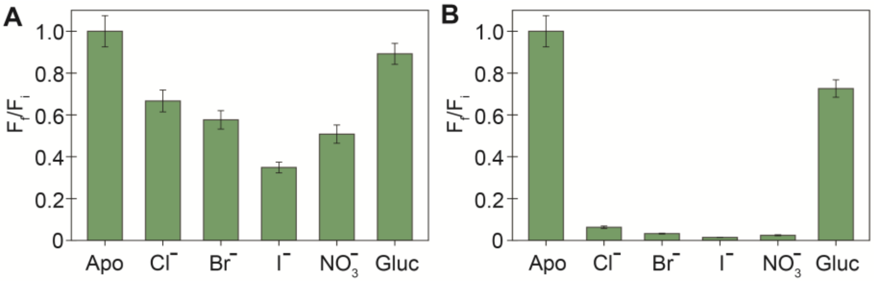

Figure S25. Normalized fluorescence response (F_f/F_i) of cgreGFP in the presence of (A) 10 mM and (B) 100 mM sodium chloride (Cl^-), bromide (Br^-), iodide (I^-), nitrate (NO_3^-), and gluconate (Gluc) (λ_{ex} = 470 nm, λ_{em} = 500 nm). Data is shown for both protein batches as the average of the three technical measurements for each batch with the propagated standard deviation.

VI. cgreGFP-H149A ion distance data from molecular dynamics simulation.

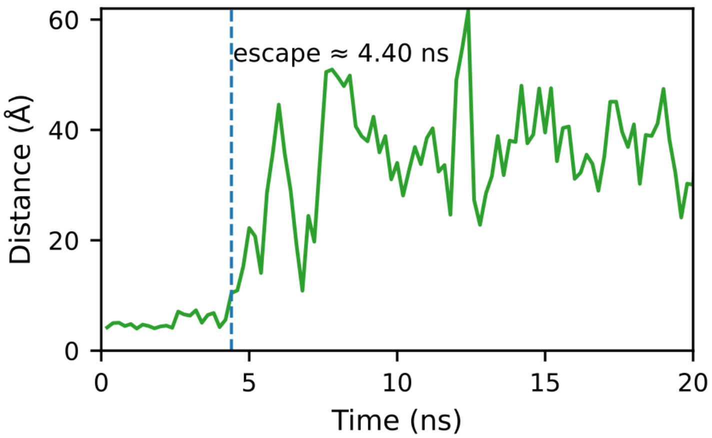

Figure S26. Distance (Å) of the chloride ion to the chromophore phenolate oxygen for the proposed H149A mutant over the first 20 ns of the cpHMD trajectory.

VI. Primers for cgreGFP-H149A.

Table S3. Primers used to introduce the H149A mutation (red) in cgreGFP.

Description	Primer Sequence (5' to 3')
Forward	ACAGCCCGCCGGCAGCGGTTTATATCC
Reverse	CGGCGGGCTGTGGTACAGCACAC

VII. cgreGFP-H149A PCR conditions.

Table S4. Polymerase chain reaction conditions (PCR) to clone the H149A mutation in cgreGFP.

Solution	Stock concentration	Volume (µL)
Template	10 ng/µL	1.0
Forward Primer	10 µM	1.5
Reverse Primer	10 µM	1.5
Phusion Master Mix	2X	12.5
DMSO	–	0.5
Autoclaved Water	–	8.0
Total	–	25

Step	Temperature (˚C)	Time (s)	Number of cycles
Denaturation	98	30	1
	98	10	30
Annealing	70	10	
Short Extension	72	360	
Long Extension	72	600	1
Storage	10	∞	1

IX. cgreGFP-H149A nucleotide and amino acid sequences.

ATGGGCAGCAGC**CATCATCATCATCATCAC**AGCAGCGGCGAGAATCTTTATTTTCAGGGC**CATATG**ACCG
CGCTGACCGAAGGCGCGAAGCTGTTCGAGAAAGAAATCCCGTACATTACCGAGCTGGAAGGTGATGTGGA
AGGCATGAAGTTTATCATTAAGGGTGAAGGTACCGGTGATGCGACCACCGGTACCATCAAGGCGAAATAT
ATTTGCACCACCGGTGATCTGCCGGTGCCGTGGGCGACCATCCTGAGCAGCCTGAGCTACGGTGTTTTCT
GCTTTGCGAAATATCCGCGTCACATTGCGGATTTCTTTAAGAGCACCCAGCCGGATGGTTACAGCCAAGA
CCGTATCATTAGCTTCGATAACGACGGCCAGTATGACGTGAAGGCGAAAGTTACCTACGAAAACGGTACC
CTGTATAACCGTGTGACCGTTAAGGGTACCGGCTTTAAAAGCAACGGTAACATCCTGGGCATGCGTGTGC
TGTACCACAGCCCGCCG**GCA**GCGGTTTATATCCTGCCGGATCGTAAGAACGGTGGCATGAAAATTGAGTA
CAACAAGGCGTTCGACGTTATGGGTGGCGGTCACCAGATGGCGCGTCACGCGCAATTTAACAAACCGCTG
GGCGCGTGGGAGGAAGATTACCCGCTGTATCACCACCTGACCGTGTGGACCAGCTTCGGTAAAGATCCGG
ACGATGACGAGACCGACCACCTGACCATCGTGGAAGTTATTAAGGCGGTTGACCTGGAGACCTATCGT**TA**
A

MGSS**HHHHHH**SSGENLYFQG**HM**TALTEGAKLFEKEIPYITELEGDVEGMKFIIKGEGTGDATTGTIKAKY
ICTTGDLPVPWATILSSLSYGVFCFAKYPRHIADFFKSTQPDGYSQDRIISFDNDGQYDVKAKVTYENGT
LYNRVTVKGTGFKSNGNILGMRVLYHSPP**A**AVYILPDRKNGGMKIEYNKAFDVMGGGHQMARHAQFNKPL
GAWEEDYPLYHHLTVWTSFGKDPDDDETDHLTIVEVIKAVDLETYR*****

Figure S27. Nucleotide (top panel) and amino acid (bottom panel) sequences of cgreGFP-H149A. The in-frame N-terminal polyhistidine tag, NdeI restriction enzyme site, mutation site, and stop codon (*) are in purple, gray, blue, and red colored font, respectively.

X. H149A purification and spectroscopy.

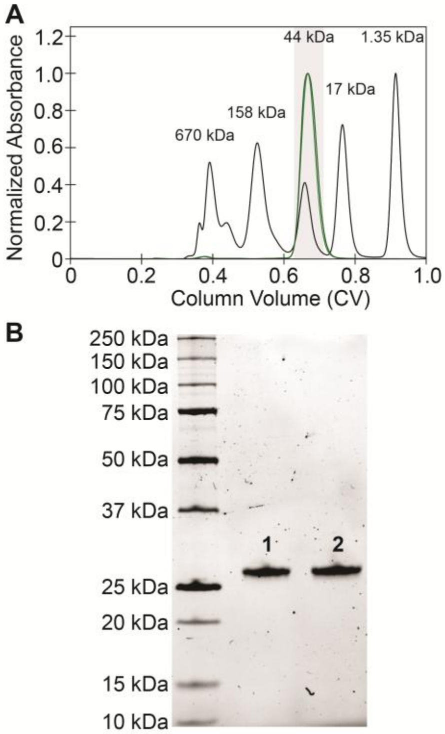

Figure S28. (A) Size exclusion chromatography (SEC) chromatogram for the first batch of *cgre*GFP-H149A (dark green), second batch of cgreGFP-H149A (green), and gel filtration standard (black) in 20 mM Tris buffer at pH 7.5 with 150 mM NaCl. All spectra are normalized to the absorption at 280 nm. The molecular weight of each protein standard is labeled on the corresponding peaks. For each cgreGFP batch, the fraction collected for further analysis is denoted in the gray region. (B) Stain-free SDS-PAGE for purified batches of cgreGFP. From left to right: protein ladder, first protein batch (1), and second protein batch (2). The theoretical molecular weight is ~28.4 kDa.

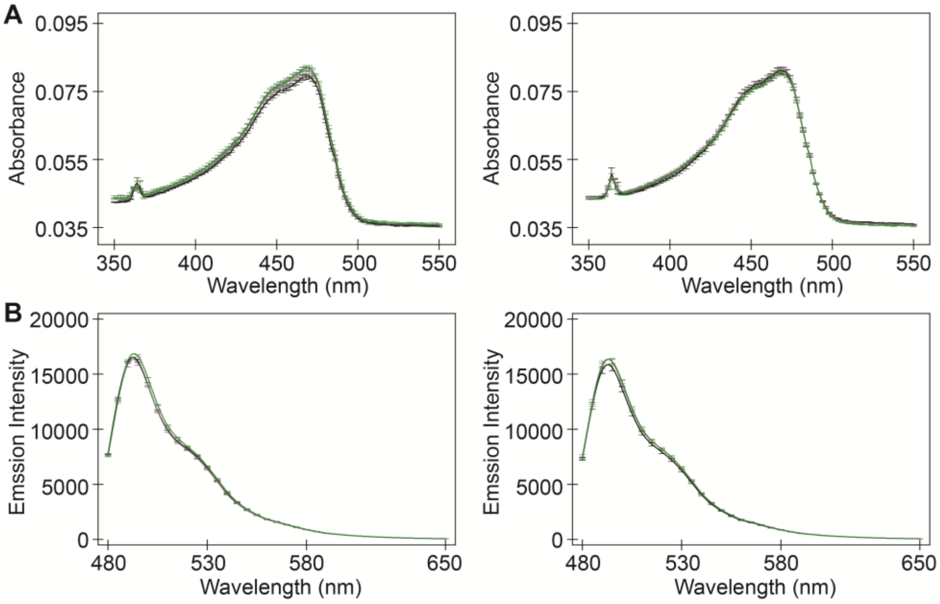

Figure S29. (A) Absorption and (B) emission spectra of cgreGFP-H149A with excitation provided at 470 nm with 0 (black), 10 mM (gray), and 100 mM (green) sodium chloride in 20 mM sodium citrate at pH 5. Data is shown for the first (left panel) and second (right panel) protein batch as the average of three technical measurements with the standard deviation.

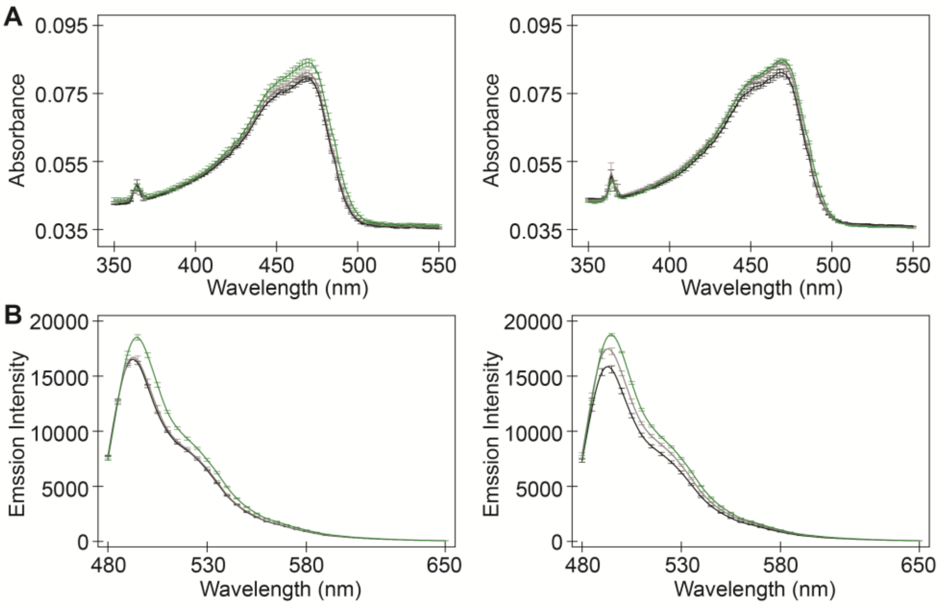

Figure S30. (A) Absorption and (B) emission spectra of cgreGFP-H149A with excitation provided at 470 nm with 0 (black), 10 mM (gray), and 100 mM (green) sodium bromide in 20 mM sodium citrate at pH 5. Data is shown for the first (left panels) and second (right panels) protein batch as the average of three technical measurements with the standard deviation.

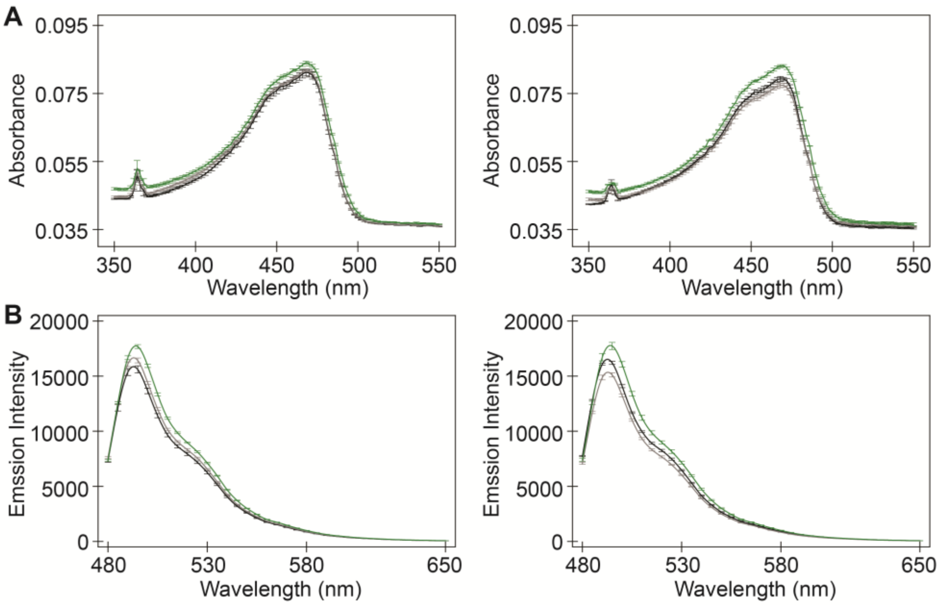

Figure S31. (A) Absorption and (B) emission spectra of cgreGFP-H149A with excitation provided at 470 nm with 0 (black), 10 mM (gray), and 100 mM (green) sodium iodide in 20 mM sodium citrate at pH 5. Data is shown for the first (left panels) and second (right panels) protein batch as the average of three technical measurements with the standard deviation.

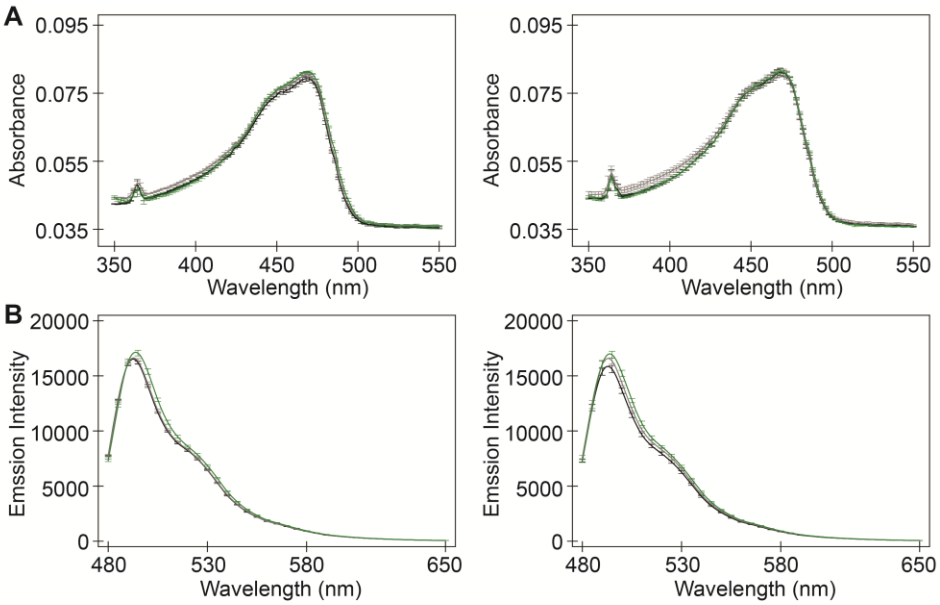

Figure S32. (A) Absorption and (B) emission spectra of cgreGFP-H149A with excitation provided at 470 nm with 0 (black), 10 mM (gray), and 100 mM (green) sodium nitrate in 20 mM sodium citrate at pH 5. Data is shown for the first (left panels) and second (right panels) protein batch as the average of three technical measurements with the standard deviation.

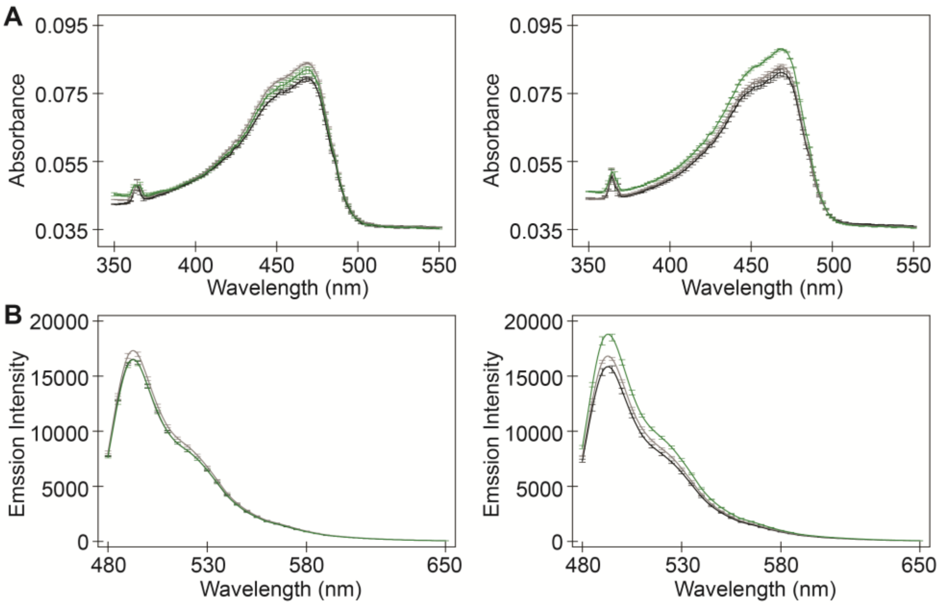

Figure S33. (A) Absorption and (B) emission spectra of cgreGFP-H149A with excitation provided at 470 nm in the presence of 0 (black), 10 mM (gray), and 100 mM (green) sodium gluconate in 20 mM sodium citrate at pH 5. Data is shown for the first (left panels) and second (right panels) protein batch as the average of three technical measurements with the standard deviation.

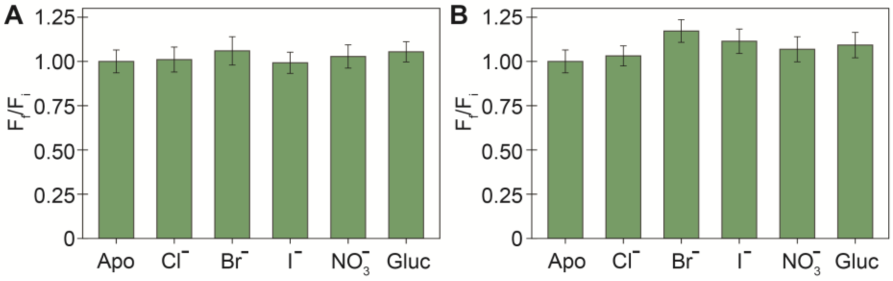

Figure S34. Normalized emission response (F_f/F_i) of cgreGFP-H149A nm in the presence of (A) 10 mM and (B) 100 mM sodium chloride (Cl^-), bromide (Br^-), iodide (I^-), nitrate (NO_3^-), and gluconate (Gluc) (λ_{ex} = 460 nm, λ_{em} = 495 nm). Data is shown for both protein batches as the average of the three technical measurements for each batch with the propagated standard deviation.

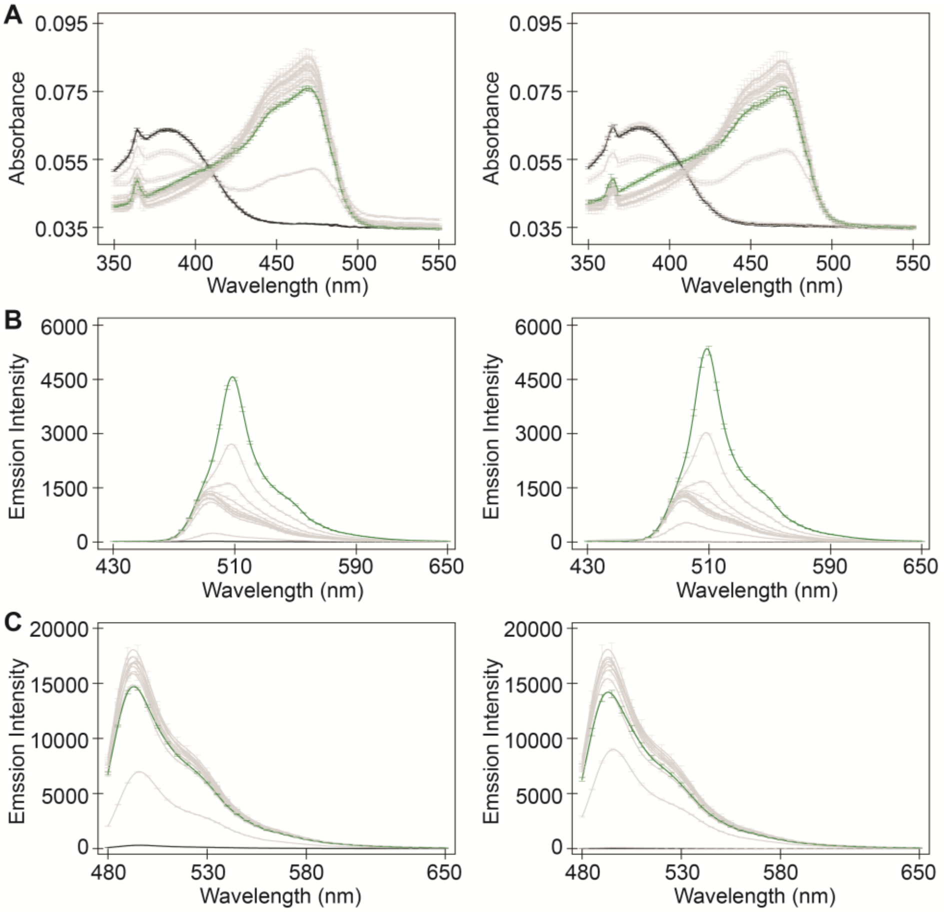

Figure S35. (A) Absorbance and emission spectra of cgreGFP-H149A with excitation provided at (B) 384 nm and (C) 460 nm in the absence (left panels) and presence (right panels) of 100 mM sodium chloride are shown from pH 3 (black) to pH 8 (green). Data is shown for the first protein batch as the average of three technical measurements with the standard deviation.

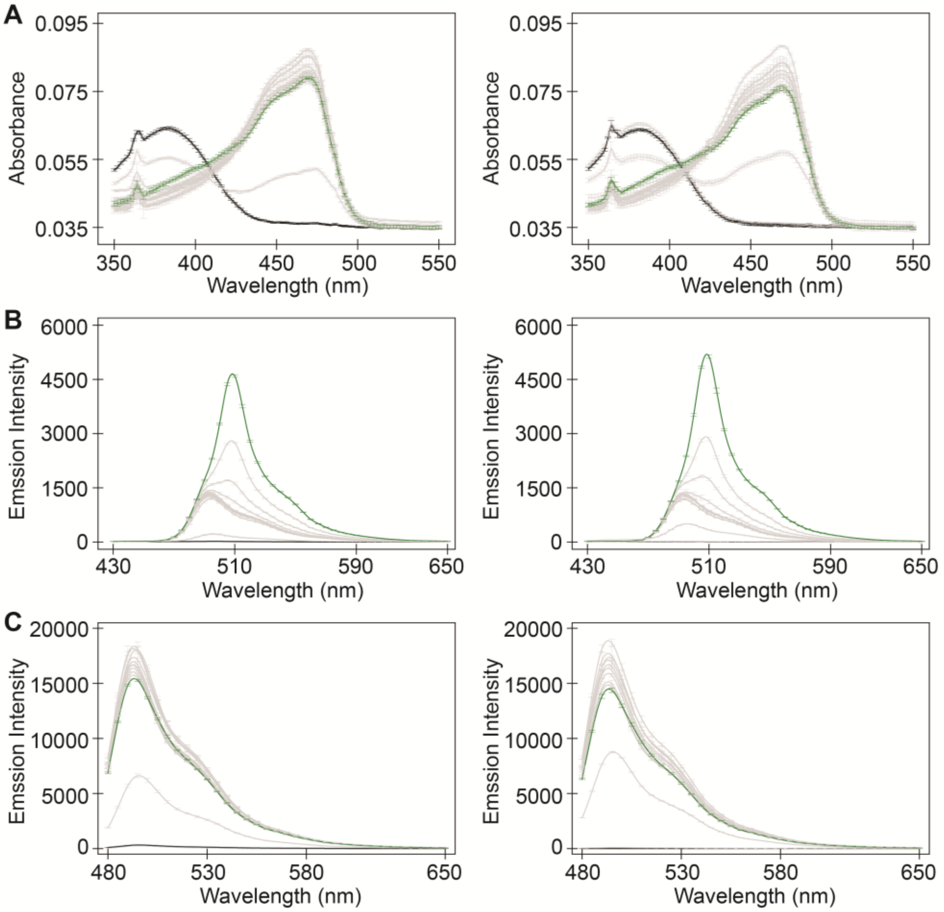

Figure S36. (A) Absorbance and emission spectra of cgreGFP-H149A with excitation provided at (B) 384 nm and (C) 460 nm in the absence (left panels) and presence (right panels) of 100 mM sodium chloride are shown from pH 3 (black) to pH 8 (green). Data is shown for the second protein batch as the average of three technical measurements with the standard deviation.

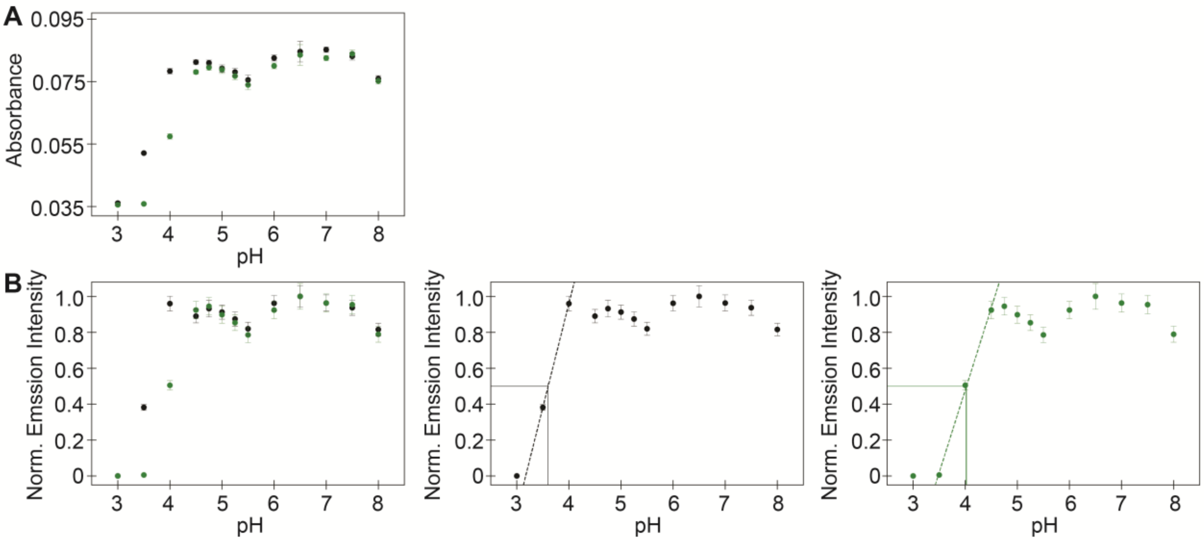

Figure S37. (A) The maximum absorbance intensity (λ_{abs} = 470 nm) plotted versus the pH in the absence (black) and presence of 100 mM sodium chloride (green). (B) The normalized emission response in the absence (black) and presence of 100 mM sodium chloride (green) plotted versus the pH (left panel) (λ_{ex} = 460 nm, λ_{em} = 495 nm). The estimated pK_a of cgreGFP in the absence (3.6, middle panel) and presence of 100 mM sodium chloride (4.0, right panel) at which 50% of the fluorescence signal is retained. Data is shown for the first protein batch.

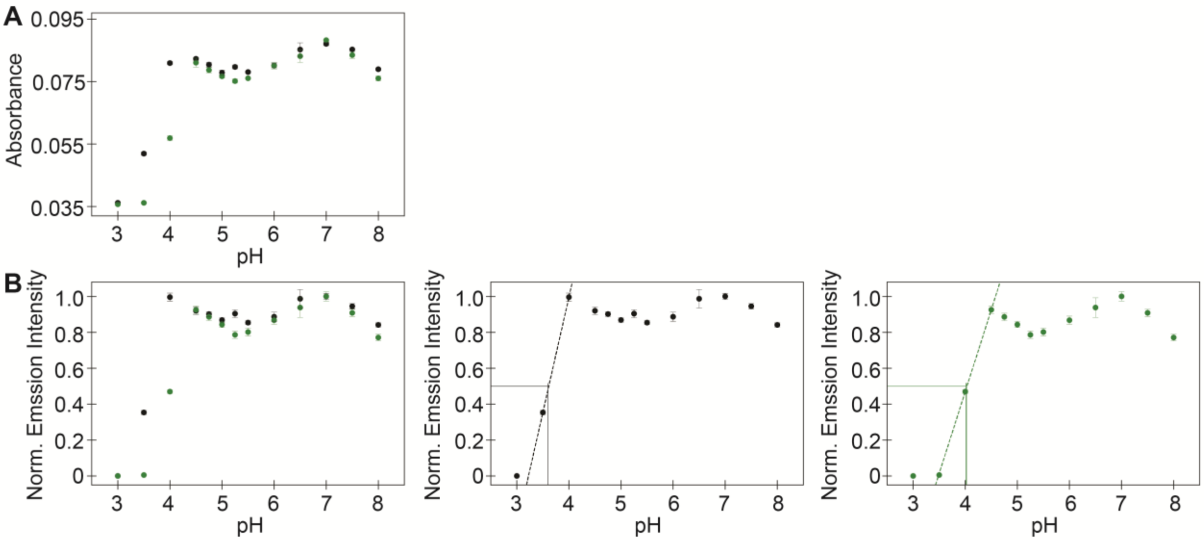

Figure S38. (A) The maximum absorbance intensity (λ_{abs} = 470 nm) plotted versus the pH in the absence (black) and presence of 100 mM sodium chloride (green). (B) The normalized emission response in the absence (black) and presence of 100 mM sodium chloride (green) plotted versus the pH (left panel) (λ_{ex} = 460 nm, λ_{em} = 495 nm). The estimated pK_a of cgreGFP in the absence (3.6, middle panel) and presence of 100 mM sodium chloride (4.0, right panel) at which 50% of the fluorescence signal is retained. Data is shown for the second protein batch.

XI. List of files from simulations included in Zenodo database.

Table S5. Files included from constant pH molecular dynamics (CpHMD) simulations via Zenodo (DOI:10.5281/zenodo.15023056).

	Filename	Description
	Forcefield Parameters	Parameters for chromophore and nitrate ion
Chloride	2hpw_cl.solv12.mod1.parm7	Topology for chloride in CgreGFP system
	2hpw_cl.solv12.rst7	Contains initial coordinates and velocities of CgreGFP with chloride for CpHMD
	2hpw_cl.solv12.cpin	Cpin input file specifying the titratable residues, initial protonation states their parameters for constant pH molecular dynamics simulation for chloride.
Bromide	2hpw_br.solv12.mod1.parm7	Topology for bromide in CgreGFP system
	2hpw_br.solv12.rst7	Contains initial coordinates and velocities of CgreGFP with bromide for CpHMD
	2hpw_br.solv12.cpin	Cpin input file specifying the titratable residues, initial protonation states their parameters for constant pH molecular dynamics simulation for bromide.
Nitrate	2hpw_Nitrate.solv12.mod1.parm7	Topology for nitrate in CgreGFP system
	2hpw_Nitrate.solv12.rst7	Contains initial coordinates and velocities of CgreGFP with nitrate for CpHMD
	2hpw_Nitrate.solv12.cpin	Cpin input file specifying the titratable residues, initial protonation states their parameters for constant pH molecular dynamics simulation for nitrate.
Iodide	2hpw_I.solv12.mod1.parm7	Topology for iodide in CgreGFP system
	2hpw_I.solv12.rst7	Contains initial coordinates and velocities of CgreGFP with iodide for CpHMD
	2hpw_I.solv12.cpin	Cpin input file specifying the titratable residues, initial protonation states their parameters for constant pH molecular dynamics simulation for iodide.
	Trajectory_snapshots	Snapshots of the four anions in their bound and unbound states.
	Chloride_entry.mov	Movie showing the entry of chloride ion into CgreGFP.
	CgreGFP_mutant.gif	Gif for Molecular dynamics simulation output showing that mutation of H143 in CgreGFP leading to the unbinding of the initially bound iodide ion from the disrupted pocket.
